# Using FRAM visualisations in quality improvement projects: identifying and testing strategies to improve anticoagulant use in the perioperative process

**DOI:** 10.1093/intqhc/mzaf074

**Published:** 2025-08-07

**Authors:** Nienke M Luijcks, Annelies Visser, Dave A Dongelmans, Dimmy M G van Dongen, Erin L de Graaf, Jop Groeneweg, Maarten J van der Laan, Perla J Marang-van de Mheen, FIAF Study Group, A Visser, D A Dongelmans, D M G van Dongen, E L de Graaf, I Grossmann, J Groeneweg, M J van der Laan, N M Luijcks, P J Marang-van de Mheen, V Jongkind

**Affiliations:** Department of Safety & Security Science, Delft University of Technology, Mekelweg 5, Delft, 2628 CD, The Netherlands; Department of Surgery, Amsterdam University Medical Centre, Meibergdreef 9, Amsterdam, 1105 AZ, The Netherlands; Department of Intensive Care Medicine, Amsterdam University Medical Centre, Amsterdam Public Health (APH), Meibergdreef 9, Amsterdam, 1105 AZ, The Netherlands; Department of Safety & Security Science, Delft University of Technology, Mekelweg 5, Delft, 2628 CD, The Netherlands; Department of Safety & Security Science, Delft University of Technology, Mekelweg 5, Delft, 2628 CD, The Netherlands; Department of Safety & Security Science, Delft University of Technology, Mekelweg 5, Delft, 2628 CD, The Netherlands; Unit of Cognitive Psychology, Leiden University, Wassenaarseweg 52, Leiden, 2333 AK, The Netherlands; TNO, Sylviusweg 71, Leiden, 2333 BE, The Netherlands; Department of Surgery, University Medical Centre Groningen, Hanzeplein 1, Groningen, 9713 GZ, The Netherlands; Department of Safety & Security Science, Delft University of Technology, Mekelweg 5, Delft, 2628 CD, The Netherlands

**Keywords:** quality improvement, risk management, systems science

## Abstract

**Background:**

To improve healthcare processes, gaining a thorough understanding of the work is important. The Functional Resonance Analysis Method (FRAM) is a method that can be used for this purpose by visualising how different steps in a process interact. However, little research is available on the use and feasibility of FRAM in quality improvement studies. Therefore, the objective of this study is to assess the feasibility of using FRAM visualisations in a quality improvement study to identify, formulate and test improvement strategies regarding anticoagulant use in the perioperative process in two Dutch University Medical Centres.

**Methods:**

Through multiple Plan-Do-Study-Act cycles, FRAM visualisations of work-as-imagined and work-as-done were created, which were validated and discussed with healthcare professionals through focus groups. Improvement suggestions were collected as input for improvement strategies from frontline clinicians. These strategies were tested and evaluated using questionnaires and interviews. The interviews were analysed using content analysis to further explore the value of the FRAM visualisations for identifying and employing improvement strategies.

**Results:**

The FRAM visualisations were perceived as confusing by professionals given their limited knowledge of FRAM, and it was time-intensive to identify possible improvements in the perioperative process. Using a simplified visualisation that showed the key FRAM information resulted in multiple improvement suggestions which were successfully tested as improvement strategies. The content analysis revealed three themes related to the use of FRAM: how care could be organised efficiently and safely, bringing stakeholders together to highlight the roles and responsibilities of professionals, and identifying how documentation of patient information is often scattered or incomplete.

**Conclusions:**

FRAM visualisations in quality improvement studies can provide valuable insights into the working process, which are also useful for formulating and testing improvement strategies. However, adjustments to the visualisations are necessary to enable professionals to participate in identifying improvement strategies.

## Introduction

Many quality improvement (QI) projects in hospitals aim to enhance working processes and patient outcomes to ensure efficient, effective and safe care [1, 2]. Understanding the current process is essential to identify underlying issues and design interventions. One method for this is mapping work-as-Imagined (e.g. protocols) and comparing it to work-as-done (e.g. everyday work), to gain insights into how processes can be improved. Functional Resonance Analysis Method (FRAM) was designed to provide an overview of day-to-day activities and to visualise interrelated activities within a system [3].

In healthcare, FRAM has been used to compare work-as-imagined and work-as-done, identify functions in a system, and improve or redesign clinical processes [4]. Additionally, FRAM can help envision the complexity of work-as-done [5]. However, McGill and colleagues also highlight that FRAM models can become overly complex, which could result in simplifying FRAM findings to handle visualisation and interpretation. This suggests that healthcare professionals without FRAM experience might find it difficult to understand the visualisation. Therefore, the question is whether it is feasible to use FRAM in a QI study, as clinicians and healthcare professionals involved in such studies may lack the necessary experience to fully understand and interpret FRAM visualisations.

Few healthcare studies have used FRAM in QI projects that also tested the formulated improvement strategies. One study created a work-as-done overview of oxygen prescribing in a British hospital by interviewing healthcare professionals [6], but it is unclear whether they were involved in identifying improvement strategies based on FRAM, which seems important given their knowledge of day-to-day work. Another study involved healthcare professionals through interviews and focus groups to formulate improvement strategies but did not test these strategies [7]. This highlights that FRAM is primarily used for process mapping [4], rather than subsequent process improvement or redesign. In other words, the effectiveness of FRAM in leading to feasible improvement strategies in QI studies has not been extensively studied.

Therefore, we aimed to evaluate the feasibility of incorporating FRAM in a QI study using multiple Plan-Do-Study-Act (PDSA) cycles [8], particularly if FRAM can be effectively used to identify, develop and test feasible improvement strategies in practice. Given this aim, we focused on identification of functions and improvement of the process by identifying differences between work-as-imagined and work-as-done but not pursue the later steps of FRAM assessing potential variability and its effects. We focus on anticoagulant use within the perioperative process because of the high-risk nature of a multidisciplinary process with many roles and responsibilities [9] and the possible errors concerning anticoagulant medication [10], thereby adding to a previous study describing the work-as-done only for preoperative anticoagulation management [11], but without identifying or testing improvement strategies.

## Methods

### Setting

The perioperative process, specifically anticoagulant medication management, was examined in cardiovascular surgery departments of two Dutch academic hospitals (H1 and H2). H1 and H2 have approximately 1.400 and 1.000 beds, and 24 and 25 operating rooms, respectively. Informed consent was obtained from all participating professionals, and ethical approval was acquired from Delft University of Technology (application 3265).

### FRAM visualisations

Work-as-Imagined and Work-as-Done regarding perioperative anticoagulant use were visualised using FRAM. A FRAM visualisation comprises interconnected functions, represented as hexagons with six couplings (Input, Output, Resource, Precondition, Time, Control) to show system relationships. Actions within the process are linked to specific roles (e.g. the surgeon makes the surgery planning), indicated by hexagon colours. Foreground functions (hexagons) directly influence other functions, whereas background functions (circles) are connected but do not generate output (e.g. surgery planning is a Resource to set the surgery date).

### PDSA-Cycles

#### PDSA-Cycle 1: describe work-as-imagined

Healthcare professionals provided documentation on perioperative anticoagulant use, including local and national guidelines. Two researchers (NL, EdG) analysed and translated these into functions and defined roles and interconnections. The FRAM was visualised using Figma software [12]. Twelve main steps in the perioperative process were identified (Appendix A) and visualised, showing twelve roles per hospital. One-hour validation sessions were organised per hospital with healthcare professionals knowing the guidelines, using Teams. Two researchers (NL, EdG) showed and explained the FRAM visualisations, which were adjusted based on provided comments. The research team decided to focus on six steps in the Work-as-Done process (Appendix A) because (i) it made it feasible to gain sufficient details on all functions; (ii) professionals noted more interactions between roles and functions in these steps, providing more room for improvement.

#### PDSA-Cycle 2: describe work-as-done

Semi-structured interviews with healthcare professionals (Appendix B) were conducted by two researchers (NL, EdG) to learn about their actions within work-as-Done. Using Teams, we interviewed eight healthcare professionals per hospital, selected based on the ten roles identified in work-as-Imagined (Table 1). Depending on the frequency in work-as-Imagined, one or two professionals per role were interviewed. Only healthcare professionals within the hospital were included. Additionally, only roles within foreground functions in work-as-Imagined were included, so the referrer, intervention specialist, consulted specialist, surgery assistant and patient were excluded. If during the interviews new roles were identified (i.e. the ward physician), these professionals were also interviewed. In H1, the pharmacist was identified but not interviewed since this was a Control background function. The interviews were recorded and then transcribed.

**Table 1.** Identified roles and interviewed healthcare professionals for work-as-done FRAM^b^

Identified in work-as-imagined	Hospital 1	Hospital 2
Referrer		
Patient		
Consulted specialist		
Surgeon	2	2
Anaesthetist	2	2
Planner	1	1
Surgery assistant		
Nursing staff	1	1
Recovery nurse	1	1
Specialist (for interventions)		
Ward Physician (WAD)	1	1
Pharmacist^a^ (WAD)		

a Pharmacist was solely identified in hospital 1.

b Per hospital is indicated which and how many healthcare professionals were interviewed per role, based on the identified roles in work-as-imagined.

Two researchers (NL, EdG) independently identified functions, roles, and interconnections for work-as-Done by analysing the interview transcripts and reached consensus. The work-As-Done layout was based on the Work-as-Imagined visualisation. Each interview produced an individual FRAM visualisation, reflecting different professional perspectives. The final work-as-Done visualisation merged all functions across individuals for each hospital. This was validated by two healthcare professionals per hospital in a one-hour Teams session. In this session, researchers first explained the FRAM, then walked through the visualisations for each step in the process. Comments made by healthcare professionals were added using Figma, and feedback on FRAM was collected through field notes. The final visualisations were discussed with the research team, deciding to focus on the first four steps of the perioperative process for the remainder of this study since most interview time covered these steps and healthcare professionals mentioned more barriers and differences from work-as-Imagined.

#### PDSA-Cycle 3: creating improvement strategies

Two focus groups (Appendix C) per hospital were conducted to discuss experiences of professionals. The first focus group aimed to gather improvement suggestions based on differences between work-as-Imagined and work-as-Done identified independently by two researchers (NL, EdG), i.e. a function present in only one visualisation, different phrasing, or a different role. The second focus group discussed the improvement suggestions in more detail, such as pros, cons and which stakeholders should be involved.

A list of participants was created together with hospital staff to ensure representation of frontline clinicians and those involved in making protocols and procedures. Already interviewed participants could also participate. We invited 7-8 healthcare professionals for each focus group per hospital (Table 2). The sessions were scheduled for 1-1.5 hours, using Teams. They were recorded and transcribed, and researchers took notes during the meeting. A detailed summary was written by the attending researchers (NL, DvD) and sent to the participants for validation.

**Table 2.** Roles involved in the focus group and improvement strategies^c^

Stages	Hospital 1	Hospital 2
Attendees focus group 1	Surgeon (2) Anaesthetist (2) Resident (1) Nursing staff (1) Pharmacologist (1)	Surgeon (1) Advisor quality (1) Internist (1) Nursing staff (1) Anaesthetist (1)^a^
Attendees focus group 2	Surgeon (2) Anaesthetist (1) Resident (1) Nursing staff (1) Pharmacologist (1)	Surgeon (1) Advisor quality (1) Internist (1) Nursing staff (1) Anaesthetist (1)
Involved in improvement strategy	Surgeon Anaesthetist Pharmacist Pharmacy assistant(s)	Surgeon Anaesthetist Pharmacist Pharmacy assistant(s)
Evaluation interview	Surgeon Anaesthetist Pharmacist Pharmacy assistant(s)	Surgeon Planner^b^

a The anaesthetist in hospital 2 could not be present during focus group 1. Therefore, an interview with the same questions of the focus group was conducted, individually. The findings of this interview were discussed in focus group 2.

b Both the anaesthetist and the pharmacy assistants were invited for an evaluation interview but chose to not reply to this invitation.

c Attendees of the focus groups, the involved roles during the testing of improvement strategies, and the conducted evaluation interviews are indicated per role and in quantity for each hospital.

#### PDSA-Cycle 4: testing improvement strategies

Based on the improvement suggestions, strategies were designed in meetings with a surgeon, a pharmacist and the research team. Strategies were based on the time and resources required to test them in practice (e.g. role of additional staff members, time spent by healthcare professionals, and technological advancements). Strategies were tested during six weeks, which was deemed sufficient to observe potential effects and gain experience with the new process. Iterative adjustments could be implemented within improvement strategies after two weeks, e.g. adding the pharmacist to a meeting with the surgeon and anaesthetist about the patient’s medication.

We collected questionnaire data and conducted open-ended interviews (Appendix D) for evaluation, both during testing and after ending the project. All professionals involved in testing the improvement strategies were invited for an interview (Table 2). All roles in H1 were interviewed, in H2 two professionals did not reply to the invitations. Interviews were recorded and transcribed.

### Outcome measures

The primary outcome constituted the experiences of healthcare professionals regarding understanding and working with FRAM, as gathered through interviews and focus groups during PDSA-cycles. This provided insights into the feasibility of employing FRAM in QI projects. The secondary outcome consisted of the identified improvement strategies and their effectiveness.

### Analyses

Experiences of healthcare professionals during validation sessions were analysed using field notes. The improvement ideas during the focus groups were tabulated. The open-ended interviews were analysed through content analysis using Atlas.ti (v24 [13]). One researcher (NL) familiarised herself with the data and coded the transcribed interviews into sentences or phrases, which were then grouped. These initial groupings were discussed with two other researchers (AV, PMvdM) and iteratively refined to reach consensus. Finally, one researcher (AV) validated the groups.

## Results

### Experiences with FRAM visualisations

The work-as-imagined and work-as-done visualisations for each hospital are presented in Appendices E and F, respectively, together with quotes supporting identified functions.

During validation sessions, healthcare professionals required additional explanations to understand FRAM visualisations before providing feedback. All participants noted that FRAM visualisations were challenging to understand and needed time to fully comprehend the large amount of visual information. Validation sessions took an hour to explain and discuss all steps, both for work-as-imagined (12 steps) and work-as-done (6 steps). Work-as-Imagined validation revealed that responsibility and taking action could not always be distinguished. The same was emphasised for work-as-done. The perioperative process documentation typically identified the main responsible party, without specifying those performing the functions.

**Figure 1.**
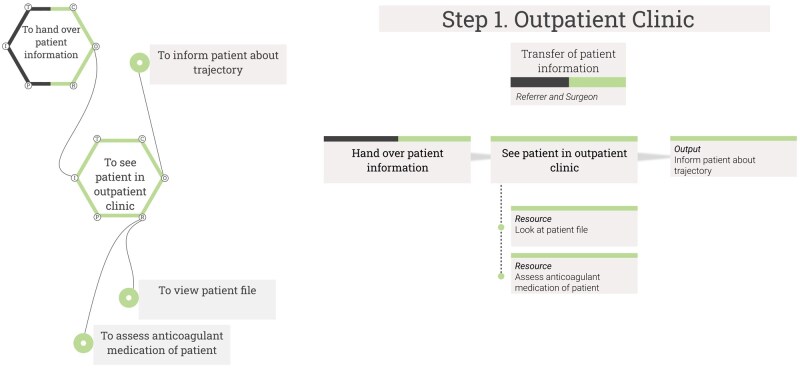
Comparison between a FRAM visualisation and the simplified visualisation. Functions of the first step of the perioperative process are visualised in FRAM (shown on the left) and functions visualised in the simplified FRAM visualisation (shown on the right) for the first step of the perioperative process. The simplified visualisation shows the process more like a flowchart, from left to right, and the FRAM visualisation is shown in a less linear manner. The step and the names of the roles are added above the steps in the simplified visualisation.

Given the time needed for explanation and the feedback received, simplified visualisations were created (NL) that preserved FRAM’s core information while omitting unnecessary aspects. To be intuitive for healthcare professionals, the simplified visualisation followed the flow of the working process. The hexagons were removed, but the *couplings* (e.g. Output to Resource) remained as grey lines. Figure 1 shows the foreground function “See patient in outpatient clinic”, with the Resource background function “Look at patient file”. A description of the main steps was added above the functions. The simplified visualisations were arranged from left to right, with the specific step and roles above each function. The complete simplified visualisations are shown in Appendix G, which were discussed during the focus groups to identify improvement suggestions.

### Improvement strategies

Table 3 outlines improvement suggestions per hospital. Multiple differences were found between the processes of both hospitals, yet there was overlap in the problems mentioned, with the same or different suggested solutions. For instance, both hospitals mentioned the patient’s incorrect anticoagulant medication but proposed different solutions (problems 5 and 6). Furthermore, some suggestions, such as hiring a case manager and EHR decision support, were unfeasible due to resource restraints.

**Table 3.** Improvement suggestions of the focus groups of both hospitals^a^

	Problem	Idea	Implementation	Responsibilities and other parties	Other solutions	Obstacles	Hospital	Connection to work process (work-as-done)
1.	Changes in anticoagulant medication between consult and admission/incorrect information in EHR	Call to check by pharmacy	Patient is called a week before surgery to check anticoagulants	Pharmacologists, but important changes should be communicated with main practitioner, planner	Real-time patient record. Check the record by pharmacologist beforehand	Elderly patients often do not know their anticoagulant medication	Both	Patients use wrong (dosage of) anticoagulant medication before admission
2.	Scattered information about anticoagulants	Add anticoagulant medication to EHR/additional training in EHR	Specific location for anticoagulants (mandatory for submission)	Physicians, nursing staff, pharmacologists. Super-users (knowledge about EPIC)	-	Permission to change EHR. Does it fit in the layout? Money.	Both	Information added to EHR by professionals cannot be found by others
3.	Preoperative screening is not done day before surgery	Check before planning preoperative screening and final surgery planning	Perform a check before patient is scheduled in surgery planning.	Planner and anaesthesiologist	Planning should use the existing checklist	You do not reach all patients, emergency patients are often not taken into account	2	Preoperative screening still has to be done at day of admission
4.	Questions in the evening about new patients	Scheduling of additional transfer moment	Earlier in the day, schedule a transfer focused on anticoagulant medication	Head practitioner, executive: ward physician, co-assistant	Real-time patient record.	Should be thoroughly documented in working agreements	1	Admission of patient to hospital day before surgery
5.	Information about patients’ anticoagulants is incorrect	Case manager for admissions	Hiring of case manager to be in contact with admissions bureau who can maintain overview of the process	Vascular surgeons. Execution could be done together with pharmacologists.	-	Case manager can be done by pharmacologists or nursing staff, but they would need extra education.	1	Information used during inpatient clinic and preoperative screening is not accurate
6.	Information about patients’ anticoagulants is incorrect	Decision-making support for anticoagulant medication	Set up different types of flow charts to make it easier for specialists to choose anticoagulant medication for the situation.	Head practitioner, anaesthesiologist, (pharmacologist assistant)	Use of existing flowcharts	This is difficult to add to the EHR-software.	2	Information used during inpatient clinic and preoperative screening is not accurate
7.	Responsibility about anticoagulants is unclear between anaesthesiologist and surgeon.	Communicate anticoagulant responsibilities clearly	Protocol mentions that surgeon is head practitioner. But not all of them decide about anticoagulants. Responsibility should be communicated more clearly.	Surgeon, anaesthesiologist, anticoagulant committee?	-	Head practitioners should keep up with knowledge about anticoagulant medication.	2	Decisions about patient’s trajectory by either surgeon or anaesthetist

a The problem discussed is described, together with an idea on how to solve the problem, how this could be implemented, who is involved and possible other solutions and obstacles. It is indicated in which hospital these points were mentioned and how it is connected to work-as-done.

The designed improvement strategy consisted of a multidisciplinary meeting (MDM) involving the surgeon, anaesthetist and pharmacist (H1) or planner (H2) a week before the surgery to address problems 2 and 5. Information about the patient’s trajectory and medication was discussed to resolve incorrect or missing information. Another shared problem concerned changes in anticoagulant medication over time (problem 1). The suggested solution was for pharmacy assistants to call the patient a week rather than a day before admission to verify and possibly correct their anticoagulant medication. This solution was incorporated into the overall improvement strategy. During six weeks of implementation, we tracked whether medication lists of patients were adjusted during the MDM (see Appendix H) to measure the impact of these improvement strategies in patient care.

Participants from H1 were more positive about the improvement strategies than H2. This was reflected in both questionnaires and interviews. Two participants in H2 did not reply to the interview invitation, and the other participants reported more barriers, while H1 identified more facilitators.

**Figure 2.**
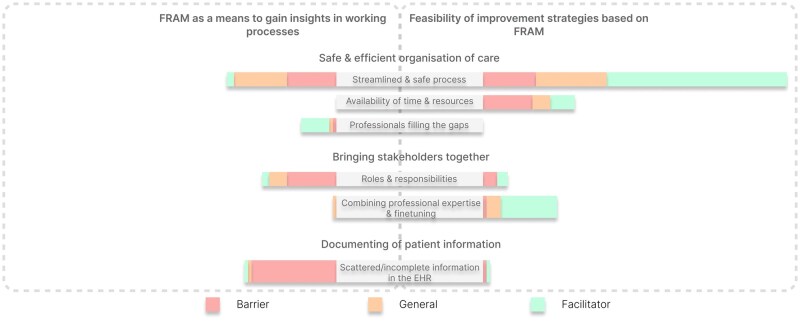
Visual representation of the content analysis. A visual overview of the amount of barriers, facilitators and general statements divided into themes (ie Safe & efficient organisation of care, bringing stakeholders together and documenting of patient information) and subthemes in the context of insight into work process or feasibility of the improvement strategy.

Content analysis revealed three themes related to the value of FRAM visualisations to identify and test improvement strategies (Figure 2, Table 4). Within each theme, FRAM provided insights into addressed barriers and facilitators in improvement strategies. The first theme, “Safe and efficient organisation of care”, highlighted how FRAM showed where professionals were filling gaps to coordinate care for patients and conducted additional checks to ensure their safety. In addition, using FRAM provided insights into how the working process was organised and how available time and resources could serve as a barrier in providing safe and efficient care. The second theme, “Bringing stakeholders together”, emphasised how using FRAM visualisations helped bringing stakeholders together by clarifying their roles and responsibilities in the working process and by sharing their expertise and perspectives when testing the improvement strategies. Finally, the third theme, ‘Documentation of patient information’, showed how using FRAM identified the currently scattered documentation of patient information in the EHR rather than consistent in the same place. Consequently, the information is often incomplete or outdated, resulting in additional checks and barriers.

**Table 4.** Content analysis findings^a^

		FRAM as a means to gain insights in working processes			Feasibility of improvement strategies based on FRAM			
		Barrier	Facilitator	General	Barrier	Facilitator	General	Quotes
*Safe & Efficient organisation of care*	*Streamlined & Safe Process*	14	2	15	15	51	20	**B:** In practice, it often turns out that the patient themselves doesn’t fully know what has been agreed upon, for example, because there is a significant amount of time between when the patient is spoken to in the outpatient clinic and when they are scheduled. (Planner, H2) **F:** The right timing with the multidisciplinary meeting and co-responsibility ensures that other check moments become unnecessary. (Surgeon, H1)
	*Availability of Time & resources*	0	0	0	14	7	5	**B:** If you want to make this standard, you also need to allocate time for it. Right now, we’ve done it sporadically because we just happened to run into each other. And whenever you institutionalize something, you need to take that into account in the planning. And then it’s no longer just two or three people doing it the same way. (Anaesthetist, H1) **F:** When it was done the day before, it felt like “that still needs to be done, and then I can go the next day.” But now they had the feeling that we are part of the process and that some time is actually being taken for us, instead of quickly entering the medication list. (Pharmacy assistant, H1)
	*Professionals filling the gaps*	1	8	1	0	0	0	**F:** These issues also come to light because I encounter them while scheduling the patient. (Planner, H2) **B:** If in doubt, we inquire, which of course leads to many actions. (Planner, H2)
*Bringing stakeholders together*	*Roles & responsibilities*	14	2	5	4	3		**B:** Letters from our department are still being sent from planners to patients. If we have a different plan than the planner, incorrect information is sent to the patient. (Surgeon, H1) **F:** In principle, we [planners] are not part of the responsibility loop, I think, but we do have a sense of responsibility in the sense that we understand that if this isn’t clear, it will affect the surgery. So in that sense, we do involve ourselves in it. (Planner, H2)
	*Combining professional expertise & finetuning*	0	0	1	1	16	4	**B:** We found out that the planner was keeping a close eye on things, and we realized that patient safety was not at risk. In that case, sitting together in an multidisciplinary meeting adds little value. We were sitting with an anaesthesiologist who was also searching through the records. We were all searching through the records together, and I didn’t find that useful. As far as I’m concerned, we won’t be continuing with this within the multidisciplinary meeting framework. (Surgeon, H2) **F:** Discussing the same problem together leads to better consensus. You can respond immediately. If needed, you can intervene right away; otherwise, you might need two or three emails to get it done. (Pharmacist, H1)
*Documentation of patient information*	*Scattered/incomplete information in the EHR*	24	1	1	1	1	0	**B:** The documentation around the perioperative anticoagulation policy needs improvement, and that is by far the most important thing. (Surgeon, H2) **F:** Careful thought needs to be put into this. The evaluation revealed that there is one place where both the planner and the surgeon—and perhaps even the patient through their records—can see what the agreement is about the blood thinners and stopping them before surgery. (Planner, H2)

B, barrier; F, facilitator.

a The main themes are defined on the left, after which subthemes are mentioned. It shows how often barriers, facilitators, and general statements were identified regarding gaining insight into the working process using FRAM and the feasibility of the improvement strategies based on FRAM. Quotes are described from the participants during the evaluation.

## Discussion

### Statement of principal findings

This study demonstrated the feasibility of using FRAM in a QI project. FRAM visualisations gave valuable insights in perioperative anticoagulant management, although they were perceived as confusing for healthcare professionals without prior experience. A simplified visualisation that contained FRAM’s core information resulted in the introduction of an MDM with relevant stakeholders and calling the patient earlier before surgery to verify and possibly correct anticoagulant medication. The evaluation showed 3 themes related to the use of FRAM visualisations and the feasibility of subsequent improvement strategies: clear and complete documentation, preferably in one place, clarity in roles and responsibilities of all stakeholders and care organised efficiently and safely.

### Interpretation within the context of the wider literature

The purpose and practical application of FRAM are widely discussed. Some say FRAM is designed to visualise system complexity [4, 5, 14], whereas others highlight that this could make the visualisation more difficult to understand [5, 15]. For healthcare, using ten to twenty FRAM functions is recommended [16]. However, with such restrictions, essential details can be overlooked. This highlights the balance between portraying reality and visualisation choices. We chose to narrow the scope from twelve to four steps throughout the PDSA-cycles to improve the steps where most barriers were identified and to reduce the chance of making an overwhelmingly large FRAM visualisation. Additionally, we simplified the FRAM visualisation to better suit the target group, as done previously for a patient handover process [17]. Therefore, practical application of FRAM towards different user groups, especially those lacking prior experience with FRAM, requires more guidelines and research.

FRAM is considered useful in gaining a systems perspective of a work process [3, 18]. In socio-technical systems theory [19], technology must support the organisation, but issues often surface in the interaction between technology and the organisation [20]. Our content analysis highlighted similar issues where documentation in the EHR was not reliable, which required additional checks in the process and thereby additional time and resources. Various previous studies have identified the need for improvement of EHRs [21, 22].

### Strengths & limitations

A strength of this study is its application in two different hospitals and combining the insights obtained from FRAM in PDSA cycles to improve the process. This showed that hospitals with a similar work-as-imagined could differ in work-as-done yet still faced similar problems. Additionally, healthcare professionals were systematically involved in every step of this QI study, which is not always done when using FRAM. This gained insight into the working process and clarified roles of individuals and colleagues, which can benefit quality of care [23, 24]. This underscores the importance of engaging frontline workers when aiming to improve work processes [25, 26].

A limitation of this study is that some healthcare professionals did not reply to the invitation for the evaluation interviews in H2. They might not have seen the improvement strategies as useful and did not want to spend any time on them, consistent with the lower ratings in the questionnaires. Possibly, more barriers regarding the improvement strategies could have been identified, although it is also possible that they had different reasons for not replying to the invitation (e.g. lack of time).

Another limitation is the short duration of testing the improvement strategies and thereby any conclusions about their sustainability. Nonetheless, the evaluation sessions provided useful insights into the feasibility of these strategies. One could argue that the patient perspective should be included, but presenting patients with a FRAM visualisation might not be feasible or logical. Improving the healthcare process is expected to benefit patient safety, and during the medication verification we received indirect (positive) feedback from patients. In future research, the input from patients within process redesign could receive more explicit attention.

Finally, a possible limitation could be not fully following the 4 steps of FRAM as designed by Hollnagel [3] and therefore not testing the full potential of FRAM in QI. However, the difficulty with FRAM lies in applying these theoretical steps in practice, for which guidelines are currently underspecified [27]. In this study, we aimed to analyse the use of FRAM to redesign and improve a process with an emphasis on involving the work floor. It seems reasonable to question whether the latter steps in FRAM concerning variability in a process are needed to achieve this aim or whether it suffices to visualise the process and further discuss improvements in practice. This seems supported by its main application in healthcare, describing work-as-imagined and work-as-done and gaining insights on improving the process [5].

### Implications for policy, practice, and research

Currently, there are few guidelines present on applying FRAM within QI studies, particularly on how to translate a visualisation into practical improvements. The presence of such guidelines could lead to better generalisability among FRAM studies. In addition, future research may further investigate the application of FRAM visualisations for different user groups. The alternative visualisation used in this study could also be applied in different contexts. Specifically, to research whether it is fit to use for more complex or non-linear processes.

In addition, there is little guidance on the selection of roles. Even though the pharmacist was only shown as a background function in work-as-imagined, they had a larger role in work-as-done and thus were involved throughout the improvement strategies. This suggests that background functions in FRAM visualisations can be equally important as foreground functions and should not be considered as a reason for exclusion in e.g. interviews.

Although healthcare professionals were involved throughout the entire process, participating professionals in H2 were less positive about the improvement strategies, despite the promising effect shown in adjusted patient medication lists (Appendix F). We may consider this improvement strategy as an intelligent failure [28, 29]. There were sound reasons why the improvement strategy initially seemed a good idea, but it turned out not to be helpful when tested in H2. Importantly, it did lead to valuable insights into the working process, particularly how roles and responsibilities were distributed among healthcare professionals.

## Conclusions

This QI study demonstrates the potential of FRAM visualisations to identify and discuss work-as-imagined and work-as-done regarding anticoagulant use in the perioperative trajectory. FRAM visualisations provide valuable insights into a system but can be resource-intensive and may require simplified visualisations. Guidelines for using FRAM in QI studies are needed to ensure it results in feasible and effective improvement strategies.

## Supplementary Material

mzaf074_Supplementary_Data

## Data Availability

The data underlying this article cannot be shared publicly due to the privacy of participants. The data will be shared on reasonable request to the corresponding author.
