# Supplementary material for: Using FRAM visualisations in quality improvement projects: identifying and testing strategies to improve anticoagulant use in the perioperative process
Source: Int J Qual Health Care. 2025 Aug 7;37(3):mzaf074. doi: 10.1093/intqhc/mzaf074 (PMC12419808; doi:10.1093/intqhc/mzaf074)
Supplement: mzaf074_Supplementary_Data [file mzaf074_supplementary_data.zip › Appendix.docx]

# Appendix

## Appendix A. Covered Steps of the Perioperative Trajectory

**Identified steps of the Work-as-Imagined, and selected steps for the Work-as-Done and the focus groups.**

|  | *Identified in Work-as-Imagined* | *Discussed during interviews Work-as-Done* | *Discussed during focus groups* |
| --- | --- | --- | --- |
| Step 1: Outpatient Clinic | X | X | X |
| Step 2: Preoperative Screening | X | X | X |
| Step 3: Planning | X | X | X |
| Step 4: Patient Admission | X | X | X |
| Step 5: Arrival at OR complex | X |  |  |
| Step 6: Arrival at OR | X |  |  |
| Step 7: Surgery | X |  |  |
| Step 8: End of procedure | X |  |  |
| Step 9: Patient leaves OR | X |  |  |
| Step 10: Recovery department | X | X |  |
| Step 11: Nursing department | X | X |  |
| Step 12: Discharge of patient | X |  |  |

## Appendix B. Interview guide to describe Work-as-Done

*Phase 1. Introduction*
Welcome of the participant, including a short explanation about the project and this session so that participants can give informed consent. Then follow the instructions for the interview: anticoagulants in the perioperative trajectory will be discussed. The participant is asked to imagine the most recent encounter with this situation of both themselves and their colleagues. It is specified that we are interested in how this occurs in practice, not in theory. If the participant is not actively involved in a step that is questioned, they are asked to mention this. The participant is given room for questions.

*Phase 2. The steps*For each step the following information was asked: Can you tell us how this step goes? For instance, where does this start, who does what, who had which role, who partook which step? In addition, for each step, researchers have aspects of the Work-as-Imagined visualisation as connection points written down.

Step 1. The inpatient clinic of the surgeon.
For researchers: Subtopics that could be mentioned: transfer of the referrer (e.g. GP) to specialist, deciding together with the patient.

Step 2. Preoperative screening
For researchers: stop moment 1 and evaluation.

Step 3. Planning
For researchers: information of planning goes to patient, surgeon, anaesthetist, OR and the department, stop moment 2: setting the date and checking the conditions.

Step 4. Admission of the patient
For researchers: admission check of information, ordering the patient, marking the patient, stop moment 3: check all preoperative decisions and the current state of patient.

Step 10. recovery department
For researchers: preconditions recovery department, arrival and transfer, stop moment 7: discharge recovery department, transfer of recovery to nursing department, transport to nursing department.

Step 11. Nursing department
For researchers: responsibilities of surgeon and of anaesthetist.

Specific points of attention for researchers were:

- What is being communicated between professionals?
- Distinction between responsibility and execution
- Where is information documented or saved?

*Phase 3. Summary and conclusion*
The participant is asked whether they have general feedback, tips, or additional comments about the interview and the process. Then, the participant is thanked for their time and the researchers tell that this interview is used to visualise the process using FRAM. If the participant is interested, their email address can be added to a list of professionals who are kept up to date about the project.

## Appendix C. Overview of Focus Group Topic Lists

### Focus Group 1

In preparation, images of the visualisations and a legend explaining the visualisations were sent to the attendees as preparation for the first session. During the first focus group, the Work-as-Imagined and Work-as-Done FRAMs were presented. The visualisations were designed and presented as a presentation in which voice recording was added to explain the process systematically. After this presentation, professionals were asked the following questions in group context:

- To what extent are there differences or similarities between Work-as-Imagined and Work-as-Done?
- What do these differences or similarities mean for the provided care?

In addition, there was room for professionals to mention specific parts of the Work-as-Imagined or Work-as-Done that they did not recognise in documentation or practice. Based on this group discussion, all professionals were asked to share one thing they would like to improve or change in the process, without considering possible restraints regarding this solution. Notes were made during the session by researchers to which information was added from audio recordings, which were sent to the attendees for validation before the second focus group.

Focus Group 2
During the second focus group, the suggested improvements of the first focus group were discussed in further detail. For each suggestion, the following questions were discussed:

- What problem is being assessed and why is it important?
- What is solved by implementing this?
- Are there alternatives?
- What are the possible consequences of this solution?
- Who is responsible within the hospital for implementing this?
- What are the uncertainties or obstacles with the implementation?

Finally, room for additional comments or questions was provided. Again, a report of the focus groups was sent to the attendees after the session for validation.

## Appendix D. Overview of Questionnaires and Interview Questions of the Improvement Strategies and Evaluation

### Data Collection During Improvement Strategies of Multidisciplinary Meeting

1. How often is the conclusion during the Multidisciplinary meeting that the patients anticoagulant policy should be changed? ___ of ____[number of total patients discussed]
2. In the case that the patients’ policy is changed, what information lead to this decision?
3. In the case that the patients’ policy is changed, what are the next steps?
4. Is there sufficient information available to make an estimation on the policy of the patient. Yes/No
5. In case of insufficient information, what information is missing to make an estimation?
6. Additional feedback

### Data Collection During Improvement Strategies of Medication Verification

*Questions to ask patient during telecommunication with patient by pharmacist.*

1. Are you confident that your anticoagulant medication is arranged properly?
2. Do you have additional information or questions about this verification?

*Questions to fill in by pharmacist after medication verification by pharmacist.*

1. How often did the medication list present in the electronic health record match the patient’s medication list? ___ of ___ [total number of patients called]
2. What did you do when the medication list in the electronic health record was not accurate?
3. Additional feedback.

### Questionnaires after Improvement Strategies

1. How much *extra* time did this improvement strategy take?

No extra time A lot of extra time

1 2 3 4 5 6 7

1. How much time did you spend on the following steps that were required after this improvement strategy (i.e. in the case of an adjustment of patient policy or incorrect medication list)

No extra time A lot of extra time

1 2 3 4 5 6 7

1. To what extent does this improvement strategy have added value?

No added value A lot of added value

1 2 3 4 5 6 7

1. What are the pros of this improvement strategy? [open answer]
2. What are the cons of this improvement strategy? [open answer]
3. To what extent does this improvement strategy contribute to information sharing between healthcare professionals in the perioperative trajectory?

No contribution A lot

1 2 3 4 5 6 7

1. How big do you estimate the change that you would like to continue using this improvement strategy in daily practice?

Very small Very big

1 2 3 4 5 6 7

1. In the case that you would like to continue using this improvement strategy, are there steps in the current trajectory that become redundant? [open answer]
2. Additional feedback.

### Interview Questions Improvement Strategies of Multidisciplinary Meeting

1. What collaboration of roles was most useful?
2. In what ways can this improvement strategy contribute to medication and patient safety?
3. In what way can this improvement strategy contribute to making the perioperative trajectory more efficient?
4. Do you see possible barriers for implementing this improvement strategy in daily practice?
   1. How could these be solved?
5. Do you have additional aspects of improvement on how to implement this multidisciplinary meeting in your daily work?

### Interview Questions Improvement Strategies of Medication Verification

1. In what ways can this improvement strategy contribute to medication and patient safety?
2. In what way can this improvement strategy contribute to making the perioperative trajectory more efficient?
3. Do you see possible barriers for implementing this improvement strategy in daily practice?
   1. How could these be solved?
4. How was it for the patients to be called earlier compared to previous experiences? What were the differences?
5. Do you have additional aspects of improvement on how to implement this earlier medication verification in your daily work?

### Evaluation questionnaire

Role of healthcare professional:

1) How much extra time did you spend on this improvement strategy?

No extra time A lot of extra time

1 2 3 4 5 6 7

2) How much time did you spend on the follow-up steps resulting from this improvement strategy (in case of policy change or incorrect medication list)?

No extra time A lot of extra time

1 2 3 4 5 6 7

3) To what extent did this improvement strategy add value?

No added value A lot of added value

1 2 3 4 5 6 7

4) What are the advantages of this improvement strategy?

5) What are the disadvantages of this improvement strategy?

6) To what extent does this improvement strategy contribute to the sharing of information between healthcare providers in the perioperative process:

Very little A lot

1 2 3 4 5 6 7

7) How likely are you to continue the actions of this improvement strategy in your daily work?

Very unlikely Very likely

1 2 3 4 5 6 7

8) In case this improvement strategy is implemented in daily work, are there steps in the current process that could be omitted?

9) Space for additional feedback

### Evaluation Interviews

**Multidisciplinary Meeting**

1) Which composition of the multidisciplinary meeting [hospital 1 either surgeon + anaesthetist, or addition with pharmacist; hospital 2 surgeon + anaesthetist, or addition with planner] did you find most useful?

2) In what way can this improvement strategy contribute to medication and patient safety?

3) In what way can this improvement strategy contribute to making the perioperative process more efficient?

4) Do you see any possible barriers if the multidisciplinary meeting were to be implemented as a standard in your work?

a. How could these possibly be resolved?

5) Do you have any other suggestions for how the multidisciplinary meeting could be better implemented in your daily work?

**Medication Verification**

1) In what way can this improvement strategy contribute to medication and patient safety?

2) In what way can this improvement strategy contribute to making the perioperative process more efficient?

3) Do you see any possible barriers if this medication verification plan were to be implemented as a standard in your work?

a. How could these possibly be resolved?

4) How was it for patients to be called earlier compared to previous experiences? Was there a difference?

5) Do you have any other suggestions for improvements?

## Appendix E. FRAM Work-as-Imagined of Both Hospitals

In hospital 1, this resulted in a total of 48 foreground functions and 43 background functions. In hospital 2, the Work-as-Imagined FRAM showed 47 foreground functions and 41 background functions. The twelve identified roles are: referrer, patient, anaesthetist consulted specialist, surgeon, planner, surgery assistant, nursing staff, holding employee, operating room staff, recovery nurse, and specialist (for interventions).

Hospital 1

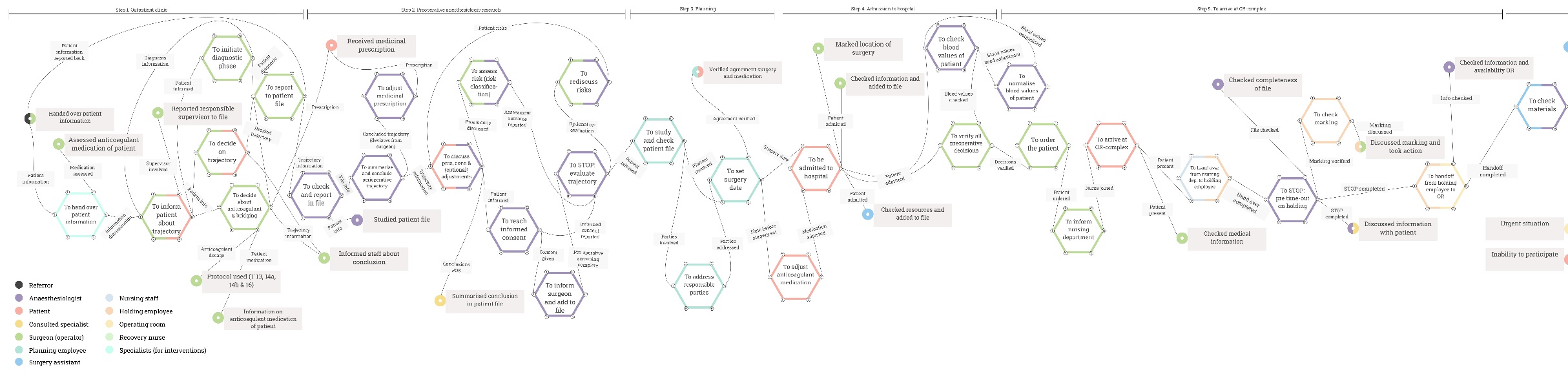

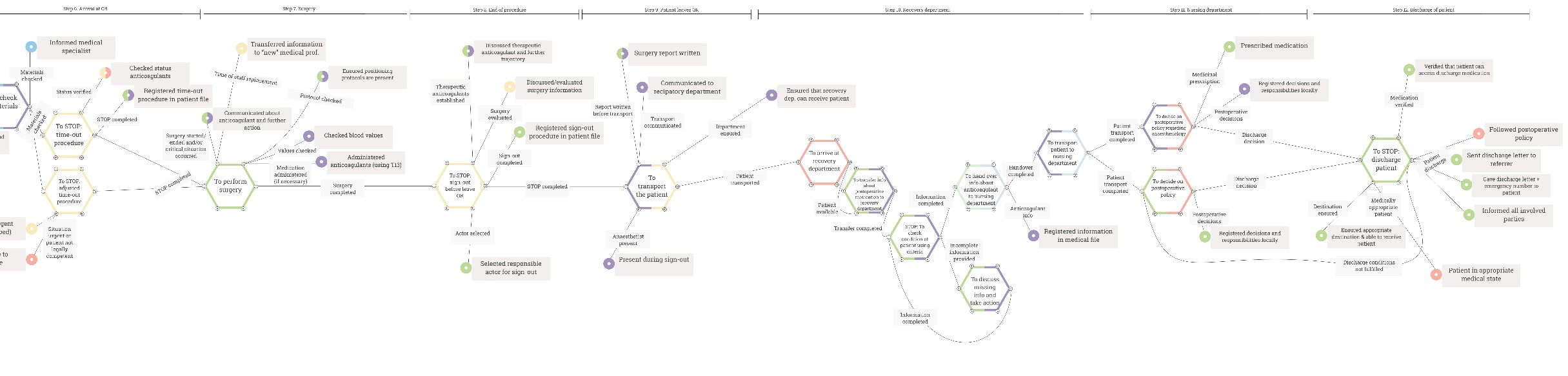


### Hospital 2


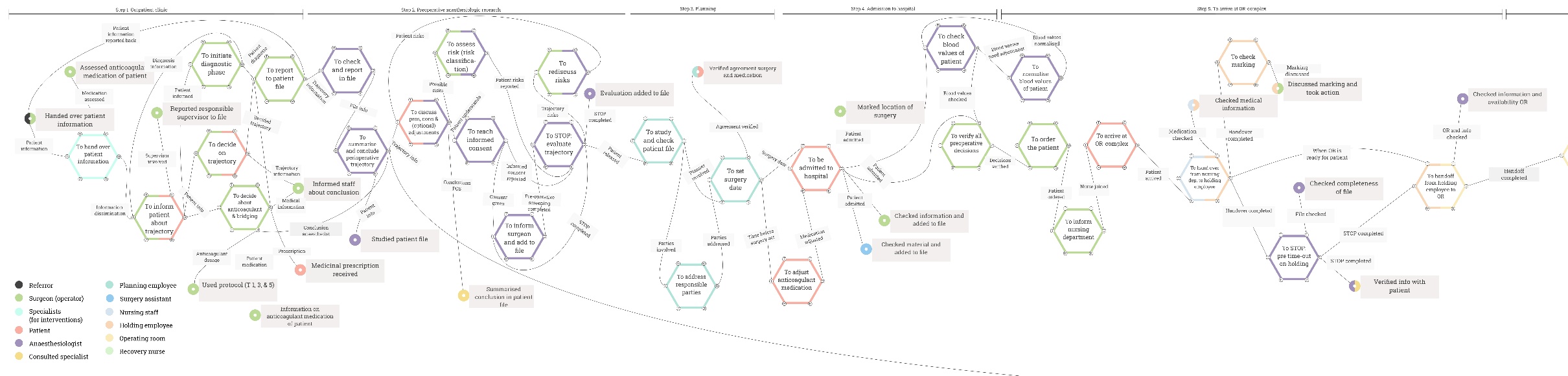


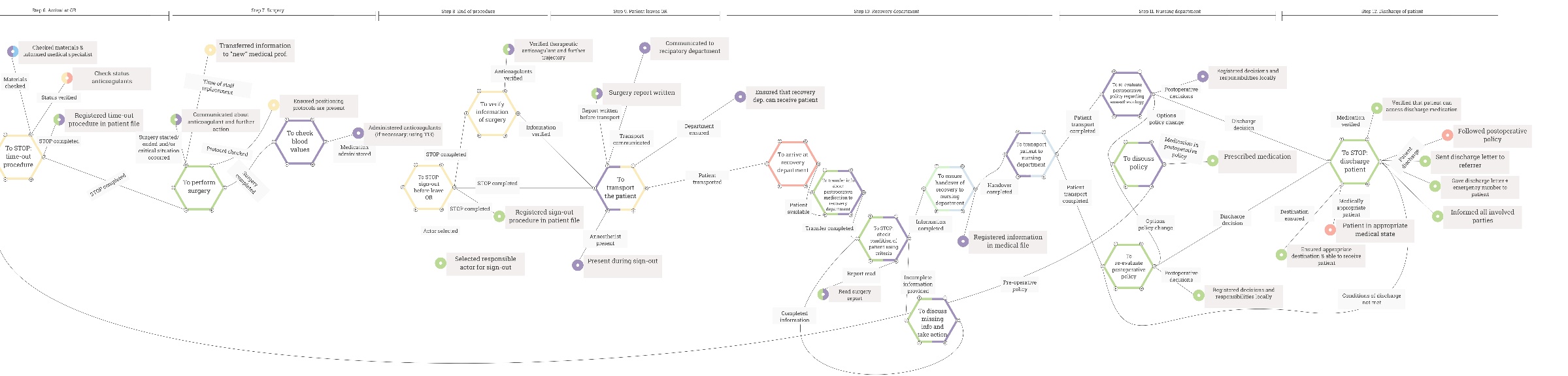


## Appendix F. Work-as-Done FRAM

In the Work-as-Done FRAM of hospital 1, 13 foreground functions and 21 background functions were identified. In addition, 8 roles were identified. These roles were the referrer, surgeon, patient, anaesthetist, planner, nursing staff, ward physician and the pharmacist. For hospital 2, this entailed 13 foreground functions, 21 background functions and 7 roles. The roles are identical to those of hospital 1, except the pharmacist was not identified

### Hospital 1.


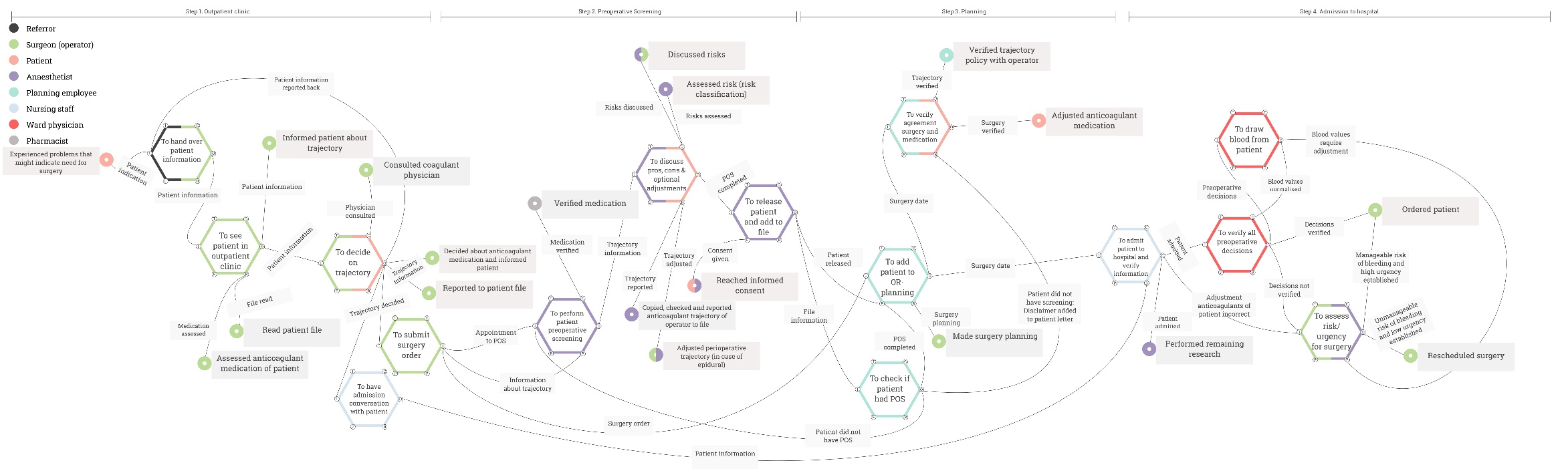


Hospital 2


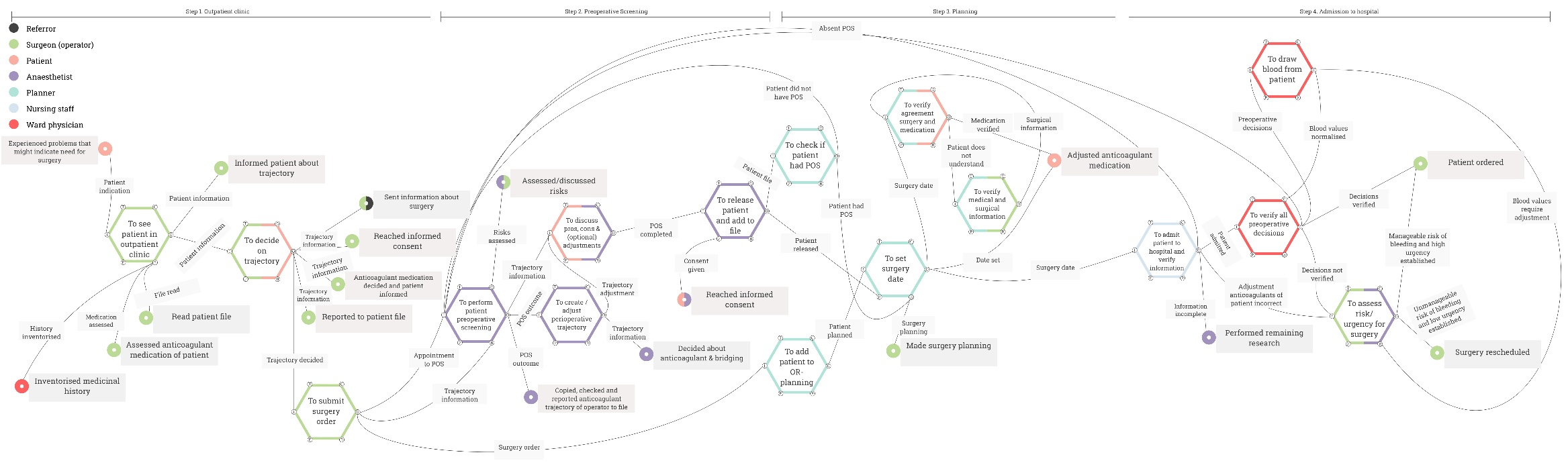


Description of Work-as-Done based on exemplary quotes from the semi-structured interviews with healthcare professionals

Note: If the hospital is not specified, the process is applicable to both hospitals.

**Step 1. Outpatient clinic**

The process starts with the *handover of patient information* from the **referrer** to surgeon.

- Surgeon: “There is often an up to date medication list with the referral of the GP”;
- Resident: “… the patient is referred to us through the GP”;
- Anaesthetist: “… patients come from the GP…”.

Afterwards, the *patient is seen at the outpatient clinic* by the surgeon. During this step, the patient is *informed about the trajectory*.

- Surgeon: “What we usually do is discuss with the patient to see what the problem is, and schedule a surgery”;
- Surgeon: “Usually the patient receives information […] on what to do”;
- Anaesthetist: “The surgeon discusses with the patient that there’s an indication for surgery and discusses everything”.

In addition, the surgeon *looks at the patient file*.

- Surgeon: “For admitted patients the EHR shows the medication they use…”;
- Surgeon: "Then you check whether that [anticoagulant medication] is somewhere in the electronic file (usually not in the right place, but it is often somewhere)."

Then the *patient’s anticoagulant information is assessed* by the surgeon.

- Surgeon: “… Regarding what they use, either we already know it from the pharmacist and usually I check with the patient whether they actually use it”;
- Surgeon: “We find it interesting whether the patient uses anticoagulant medication and if something has to happen with it before or after surgery”;
- Anaesthetist: “One of the standard aspects that has to be discussed is whether the patient uses anticoagulants, and then there should be made a plan with how to handle anticoagulants”.

[hospital 2] The *medicinal history is inventoried* by the Ward physician with the surgeon.

- Nurse: "The surgeon should inventory the home medication at the outpatient clinic. The ward doctor is the one who then takes over the file";
- Nurse: "Orders come from the ward doctor, which come from anaesthesia reports and notes that a patient brings with him. The patient is always asked to bring a recent medication overview from the pharmacy, but this simply does not happen."

Together, the surgeon and patient *decide on the trajectory*.

- Planner: "The doctor may then decide to perform an operation. He will of course discuss this with the patient and then a choice will be made";
- Surgeon: "The patient has every say, but if he wants to continue taking anticoagulation and I think it is a high risk of bleeding, then we are not going to come to an agreement together. But it never happens that a patient does not agree with it, he or she comes to you as an expert."

This includes the *decision about anticoagulants medication and informing the patient* by the surgeon.

- Anaesthetist: "In principle it is of course the surgeon's responsibility to discuss this and arrange it, so if the patient has to be clearly switched on anticoagulation, then the surgeon should also arrange that.”

The *patient is informed about the trajectory* by the surgeon *and gives consent*.

- Surgeon: “…Usually the patient receives information about admission and how to handle that”;
- Nurse: “The patient agrees with getting surgery”;
- Planner: “The surgeon can decide whether a surgery is required. Of course he discusses this with the patient and decisions are then made”;
- Anaesthetist: “The pros and cons are discussed including possible complications…”;
- Anaesthetist: “I will talk to the patient after the surgeon spoke to the patient and decided that a surgery is required”.

[hospital 1] For this, it could be that *a coagulant physician is consulted* by the surgeon.

- Surgeon: "If someone has difficult problems with all kinds of other diseases related to clotting, we discuss it with the coagulation doctor, a kind of internist";
- Surgeon: "If there is something special about the anticoagulation, we will contact the coagulation doctor."

The surgeon *submits the surgery order*.

- Surgeon: "If we put someone on the list for surgery, we have to submit a request in the EHR, which contains questions about anticoagulants;"
- Nurse: “The doctor must put an admission order in it".

The *information about the surgery trajectory is communicated* to the **referrer**

- Surgeon: "I tick that and describe it in my operation request, and almost always in my status (for the letter for the GP), and you tell the patient."

[hospital 1] Sometimes, the nurse already *has the admission conversation* with the patient.

- Nurse: “The patient has been to the doctor and we will then do the admission interview at the outpatient clinic. If we do not get around to it, it will happen on the day of admission”;
- Anaesthetist: “"If a patient is admitted, a nurse will an intake in the department or that has already happened on an outpatient basis, that is also possible, that is happening more and more, that some things have already been discussed on an outpatient basis, but if a patient is not scheduled for a very long time, it may well be that a number of things are done again have been changed and the ward doctor actually monitors the patient's status".

**Step 2. Preoperative screening**

The anaesthetist *performs the preoperative screening* on the patient.

- Anaesthetist: “Then he is forwarded to us, and then ideally additional research has already been done, the lab research has been done and if this is not the case, we will first discuss it with the patient or see the patient and then we will do additional research. In principle it should be done";
- Surgeon: “the POS (screening) is done via anaesthesia. If that screening has not been done, you cannot operate on the patient.”

[hospital 1] The pharmacist performs the *medication verification*.

- Planner: “They also have a conversation with someone from the pharmacy, so the latest medication is verified there, as well as anticoagulants.”

The *pros and cons and optional adjustments are discussed* by anaesthetist and patient.

- Anaesthetist: “Then you fill it out and ultimately you discuss with the patient what kind of anaesthesia techniques you are going to use and what the risks are if this has not yet been discussed digitally because there are very specific things associated with this patient";
- Anaesthetist: "With anticoagulation, it is always a matter of weighing the risk of thrombosis versus bleeding: how much bleeding is the maximum acceptable amount, where I can still operate, that must be determined by the surgeon."

[hospital 1] This involves a *risk classification* by the anaesthetist.

- Anaesthetist: “ "Then the preoperative risks surrounding the anaesthesia are looked at. These are the risks for difficult ventilation, difficult intubation, comorbidities, should things be excluded? Should someone be better off, are they even fit for surgery? And all actions preoperatively are also agreed upon.”

*Risks* are possibly *rediscussed* with the anaesthetist and the surgeon. In some cases, the *perioperative trajectory is adjusted* by the anaesthetist [hospital 2] and the surgeon [hospital 1].

- Anaesthetist: “I usually try to call the surgeon while the patient is still with me, because I think it is important that the patient understands what is going on and where the snags are. I think it is important that the patient understands that”;
- Anaesthetist: “It is very unusual for a surgeon to say, let's do this or that, and he or she will send the patient to the anaesthetist. Even though the surgeon is supposed to do this together, most surgeons do not feel comfortable with that. to draw up a ready-made plan regarding anticoagulation and forward it to the anaesthetists. It is extremely rare that this happens. There are of course surgeons who say: you know, that patient is on such and such medication and I cannot operate on that (ophthalmologist, or neurosurgeon). 'Just think of something about how you can bridge/reverse/delay that.'

The anaesthetist *copies, checks and reports the anticoagulant trajectory* of the surgeon *to the file*.

- Surgeon: “But they get them in advance at the outpatient clinic and they see our proposal and what they use. They then simply adopt that and adjust their anaesthesia plan accordingly. They don't do more than that."

An *informed consent* is reached between anaesthetist and patient.

- Anaesthetist: "What you actually want is a consensus of what you have discussed, sometimes, imagine that it would be best to get an epidural, but some patients say, I don't want an epidural, then of course you start the conversation about why someone who doesn't want that, you try to figure out what the reluctance is, some people who say, I just want it”;
- Anaesthetist: "Then informed consent from the patient whether he agrees or disagrees.”

The *patient is released* and this is added to the file by the anaesthetist.

- Anaesthetist: “This is an internal letter, That's just a note. What we make in the EHR.”

**Step 3. Planning**

The planner *adds the patient to the OR-planning*. This is based on the *surgery planning* from the surgeons.

- Planner: "And in the meantime we already see that the patient is on the list, and then things start to take effect, especially if you look at vascular patients”;
- Planner: “I see this coming in, and if it is correct, it will say whether someone is already taking anticoagulation or not. So if the patient has been to the surgeon and it has been decided to operate, it will be included in a list and ultimately in a schedule.”
- Surgeon: “A fellow vascular surgeon usually does the planning.”

Here, the planner *checks if the patient has had their preoperative screening*.

- Planner: "So we always check when we are going to schedule someone: has someone already been scheduled, has someone already been released? If not, or the appointment is too late, we will take action, we will contact the anaesthesiology clinic”;
- Planner: “"We call these patients and then we start digging, then we look to see whether the anaesthetist has given his approval. If that is not the case, then we will see whether it can still be arranged before the agreed date.”

The *surgery agreement is verified* by the planner with the patient, and with the surgeon.

- Surgeon: “As soon as the planning is ready, the planning office will contact patients by letter as long as the day is not very short. Then they send a letter with admission date, separate letter with anticoagulation advice and what should be stopped or not. If short notice by telephone”;
- Planner: “check whether someone uses anticoagulants or not, if someone does use them, if it is correct, also fill in what he is using, and if relevant, how many days before the procedure he should stop using it. If it has been partially filled in, we first ask the patient, If the patient knows clearly what the intention is, it is dealt with, if the patient does not know or it is different from what is stated in the system, then we ask the main practitioner, the surgeon.”

[hospital 2] If the patient does not understand, the planner *verifies medicinal and surgical information* with the surgeon.

- Planner: “If the patient knows clearly what the intention is, it is dealt with, if the patient does not know or it is different from what is stated in the system, then we ask the main practitioner, the surgeon.”

Afterwards, the patient can adjust, increase or reduce their anticoagulant medication.

**Step 4. Admission to hospital**

The *patient is admitted to the hospital* by the nurse and *information is verified*.

- Nurse: “They then go to see the nurses for an admission interview and suppose it has already happened at the outpatient clinic, it is more a matter of checking whether there are any details or whether things have changed during the waiting time, so to speak”
- Nurse: “They will be admitted by a nurse from the nursing department.”

If necessary, the anaesthetist can *perform remaining research*.

- Planner: "Then the screening has already been done, but is not yet completely finished or the patient still has an appointment on the admission day and that may be the case";
- Nurse: “If necessary, there is still the option every day to screen patients for the next day.”

The ward physician *verifies all perioperative decisions* and *draws blood from the patient*.

- Nurse: "Then they see a co-assistant and a ward physician and, in principle, also the staff call;”
- Ward physician: “We have to check the blood values, because someone cannot have certain blood values ​​before an operation.”

If these decisions are not verified, or the blood values require adjustment, the *risk for surgery is assessed* by the surgeon and anaesthetist.

- Surgeon: “Or you do it, but you correct it and you accept a slightly higher bleeding risk. Consideration of urgency;”
- Anaesthetist: “If he thinks that the operation can go ahead, but there are some ifs and buts, then it will be done in consultation with the team;”
- Anaesthetist: "Do you also discuss the risks with the patient, do you want to take that risk? The question is also whether anaesthetist wants to take that risk? Ultimately, surgeon and patient and anaesthetist make the final decision. Only if all three are willing to take the risk will the patient continue."

If the risk is too high or the urgency low, the *surgery is possibly rescheduled* by the surgeon.

- Surgeon: “So you can choose, that is not wise, we will wait and we will operate again in 2 weeks and then it must have stopped;”
- Surgeon: “The decision here whether to continue or not is made by the main practitioner;”
- Anaesthetist: “"Depends on which patient, which surgery, some only arrive on the morning of the operation, then you have to assume that everything has been arranged properly, if not, it will be cancelled.”

If all is verified, the patient is ordered by the surgeon.

## Appendix G. Alternative visualisations

### Hospital 1.

#### Work-as-Imagined


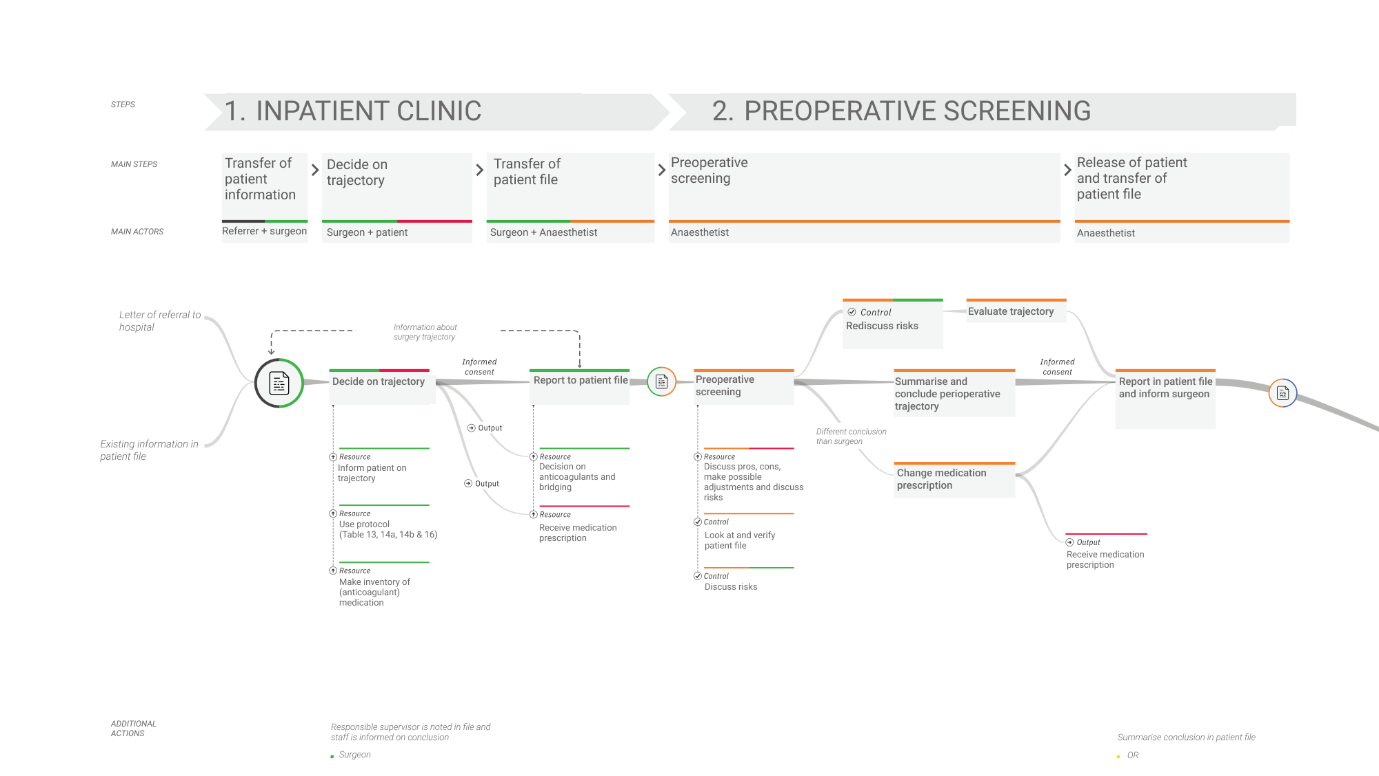


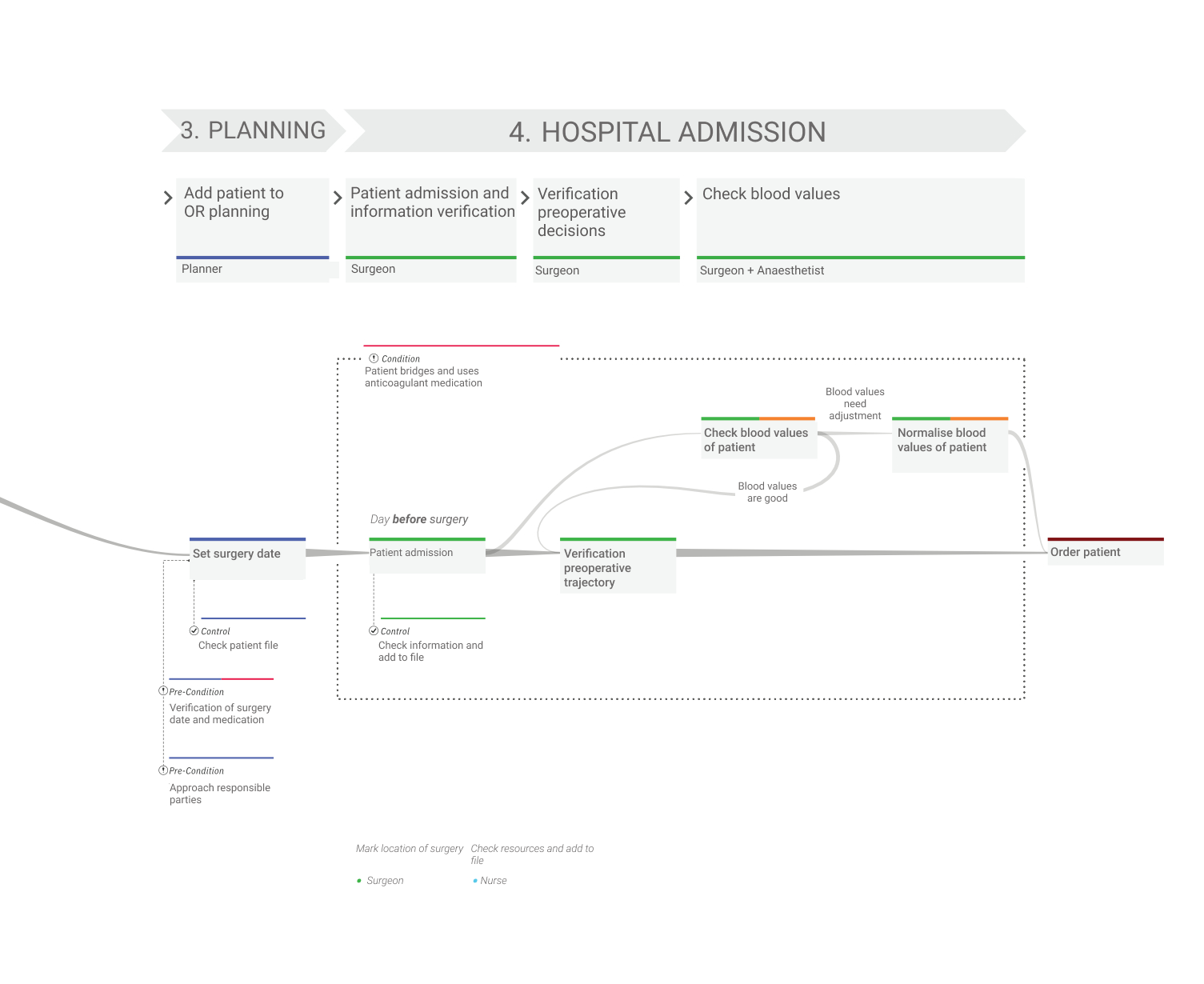


#### Work-as-Done


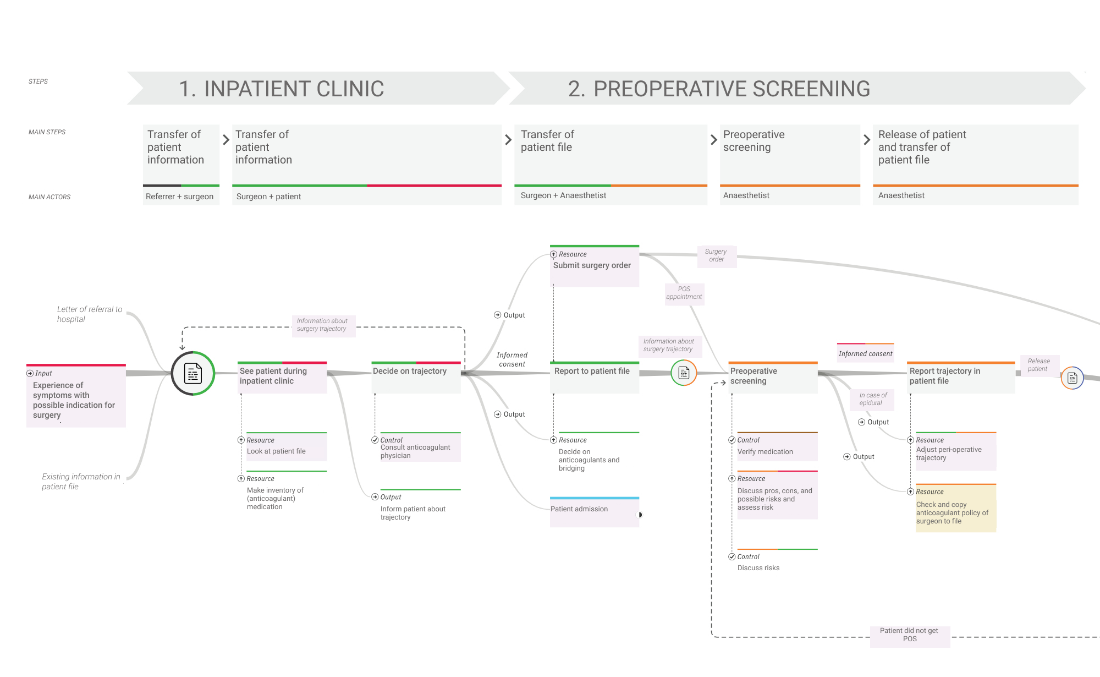


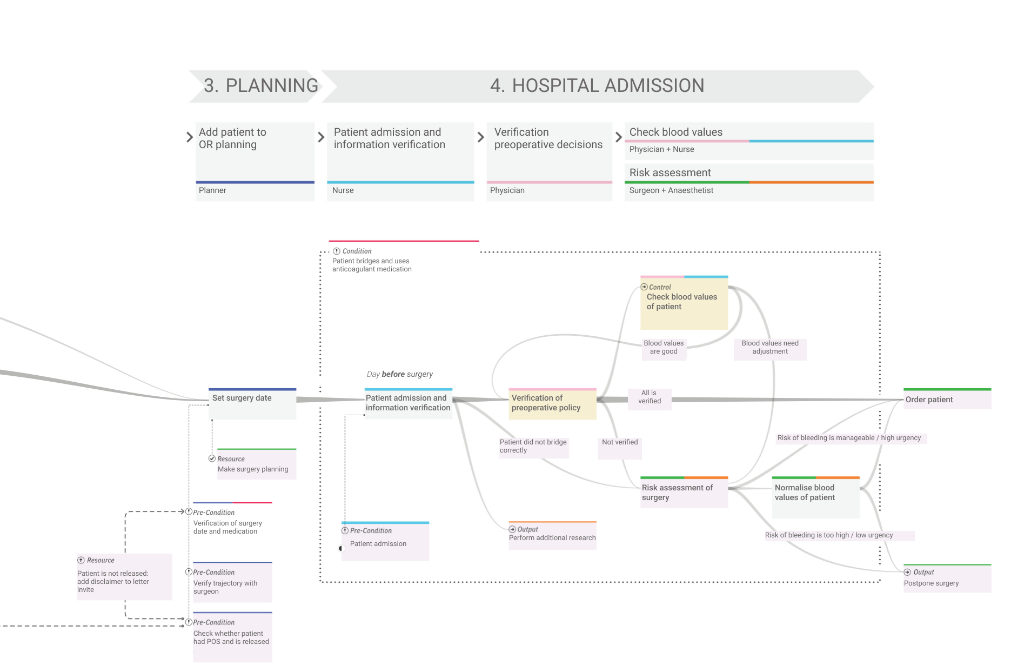


### Hospital 2.

#### Work-as-Imagined


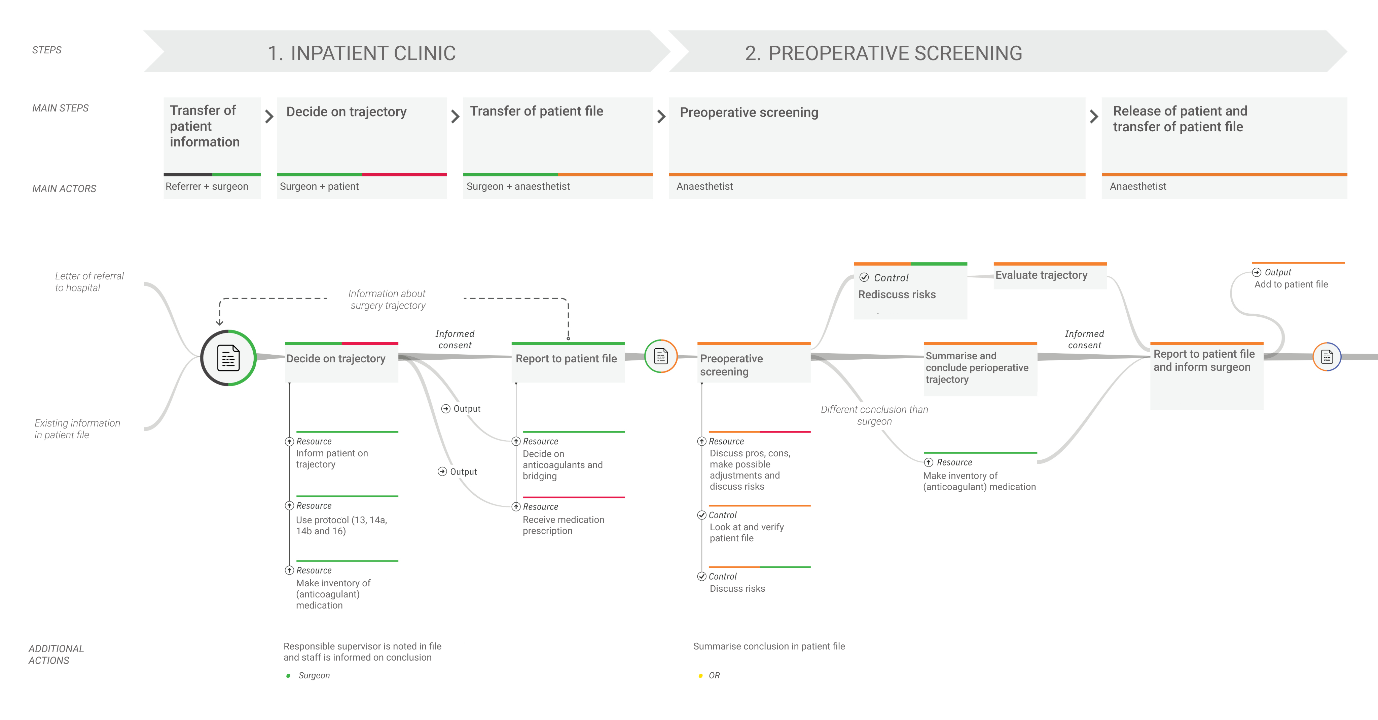


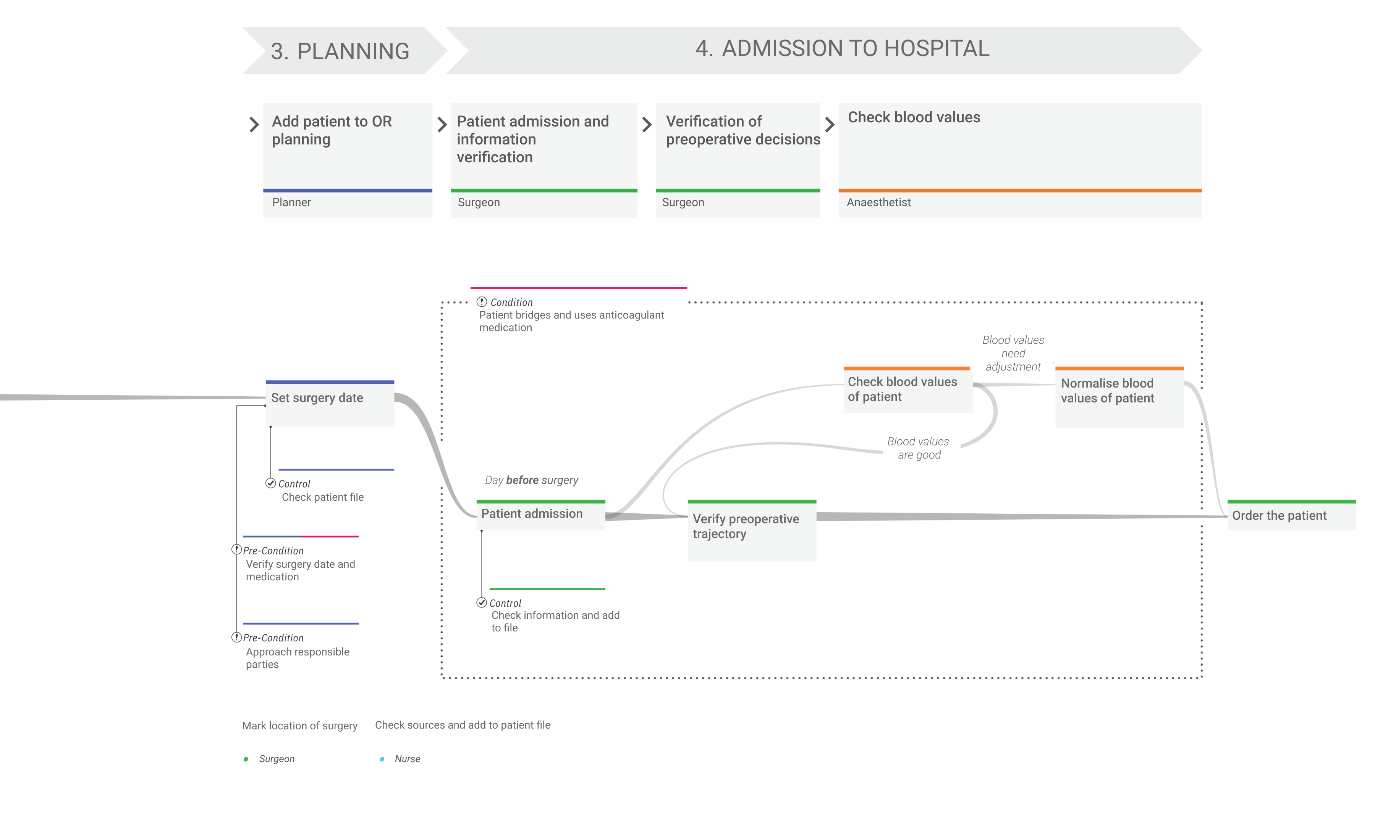


Work-as-Done


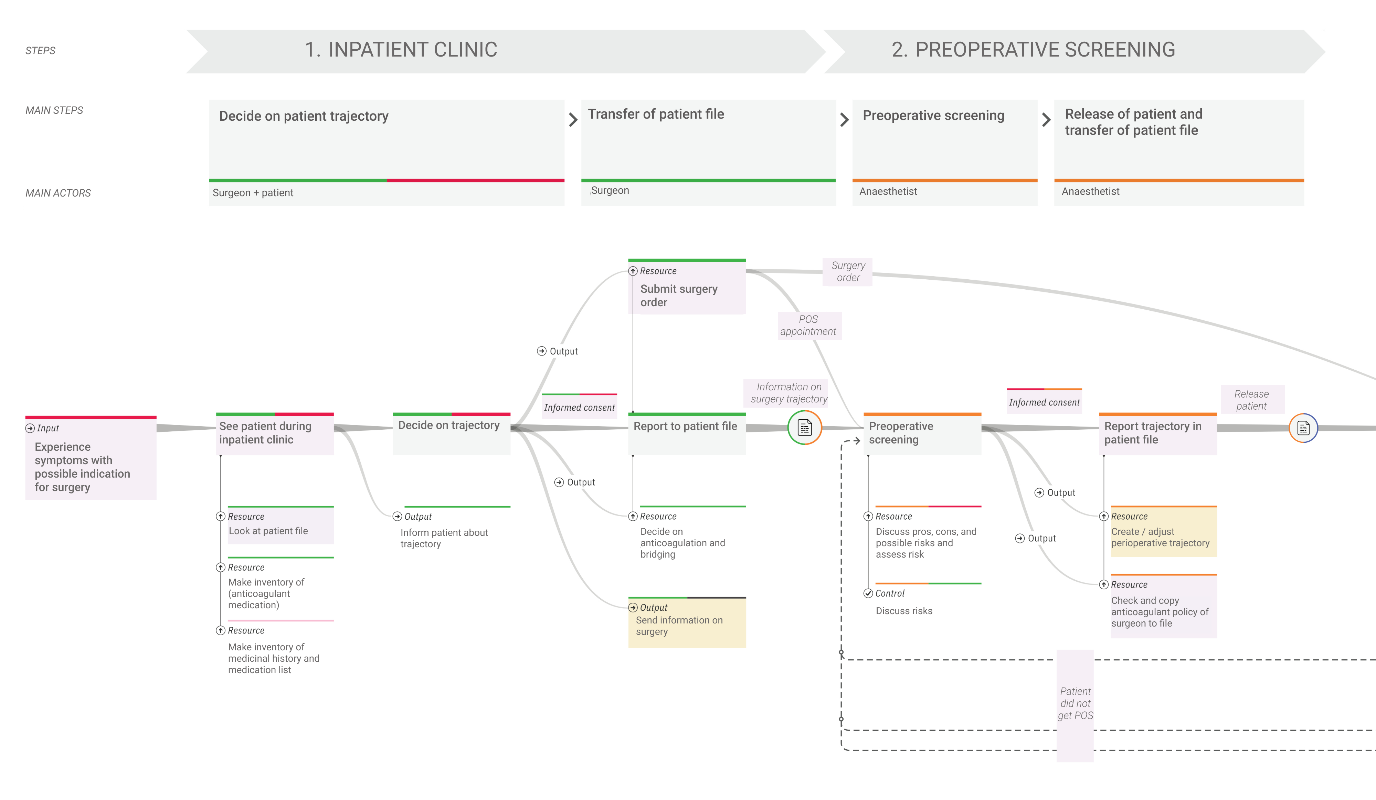


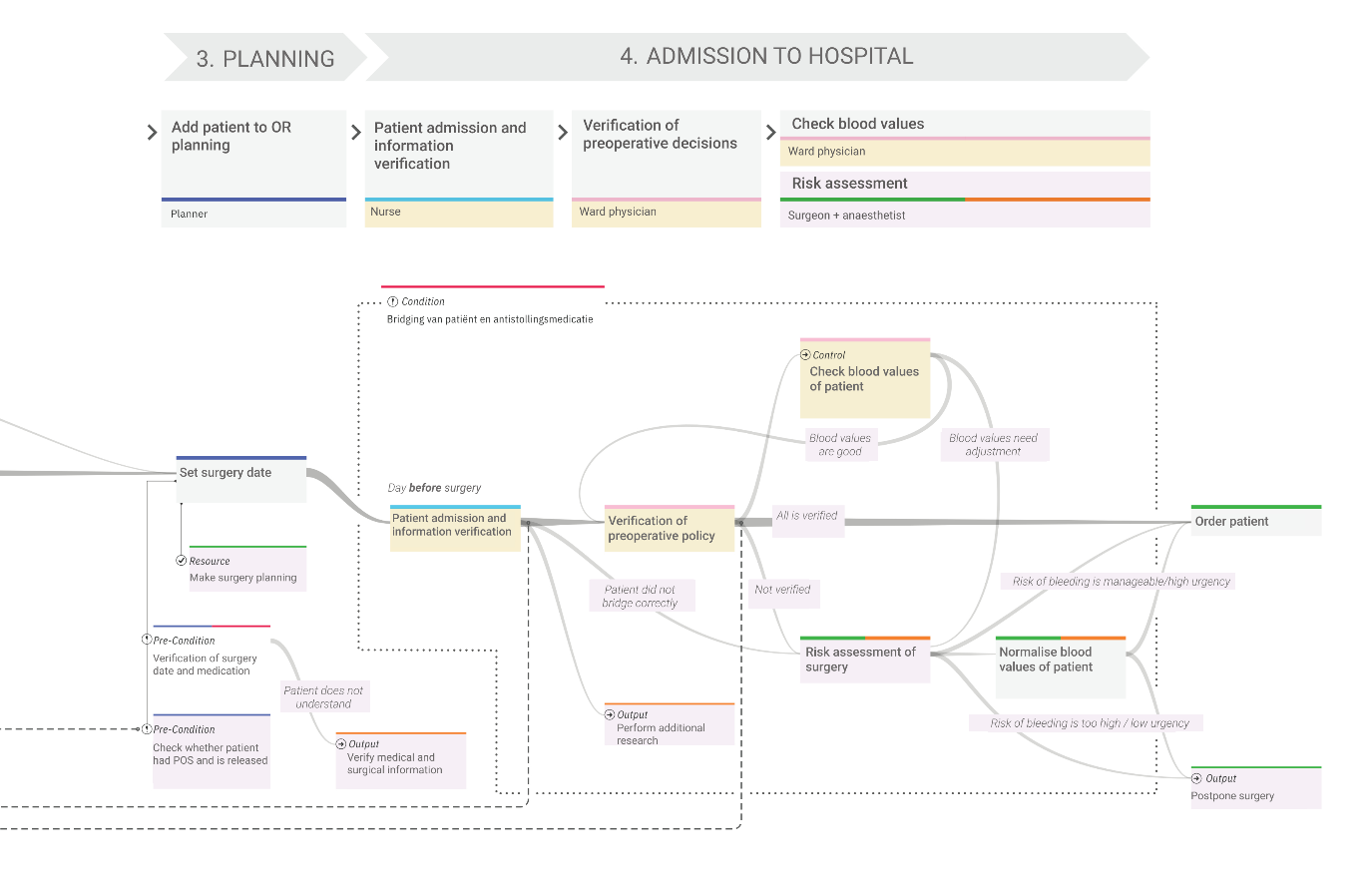


# Appendix H: Results of the Improvement Strategies

**Supplementary Table 1.**

|  | **Patients discussed** | **Policy of patient changed** |
| --- | --- | --- |
| H1 | 3 | 1 |
|  | 5 | 0 |
|  | 5 | 0 |
|  | 7 | 2 |
|  | 5 | 0 |
|  | 6 | 1 |
| H2* | 10 (4) | 4 |
|  | 8 (4) | 0 |
|  | 13 (9) | 1 |

* In hospital 2, it was documented which patients were using anticoagulant medication. For instance, 10 patients were discussed, of which (4) were using anticoagulants.
