# Supplementary figures and images for: Using FRAM visualisations in quality improvement projects: identifying and testing strategies to improve anticoagulant use in the perioperative process
Source: Int J Qual Health Care. 2025 Aug 7;37(3):mzaf074. doi: 10.1093/intqhc/mzaf074 (PMC12419808; doi:10.1093/intqhc/mzaf074)

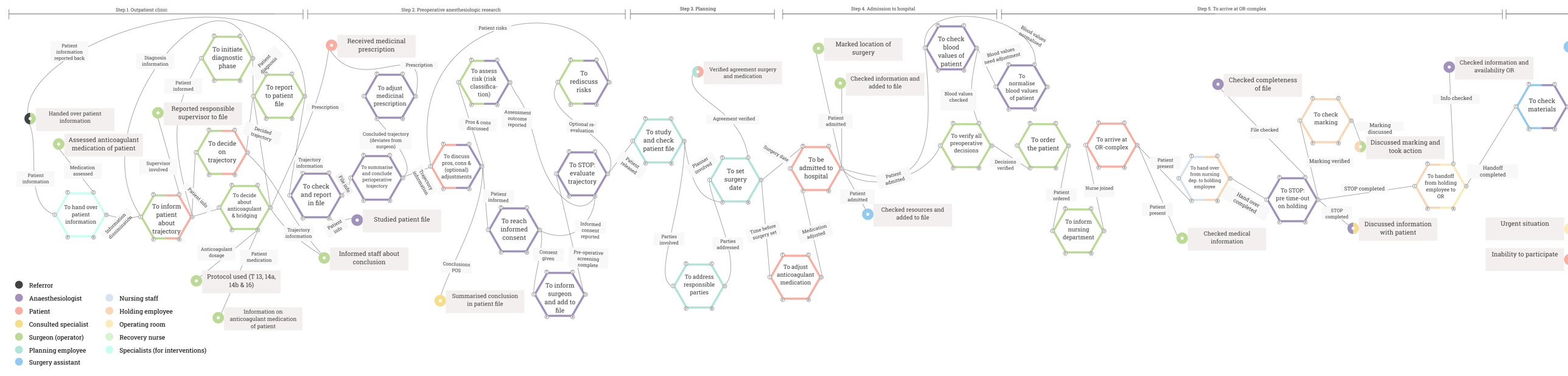

Supplement: mzaf074_Supplementary_Data [file mzaf074_supplementary_data.zip › Appendix E. Hospital 1 WAI_1_page-0001.jpg]

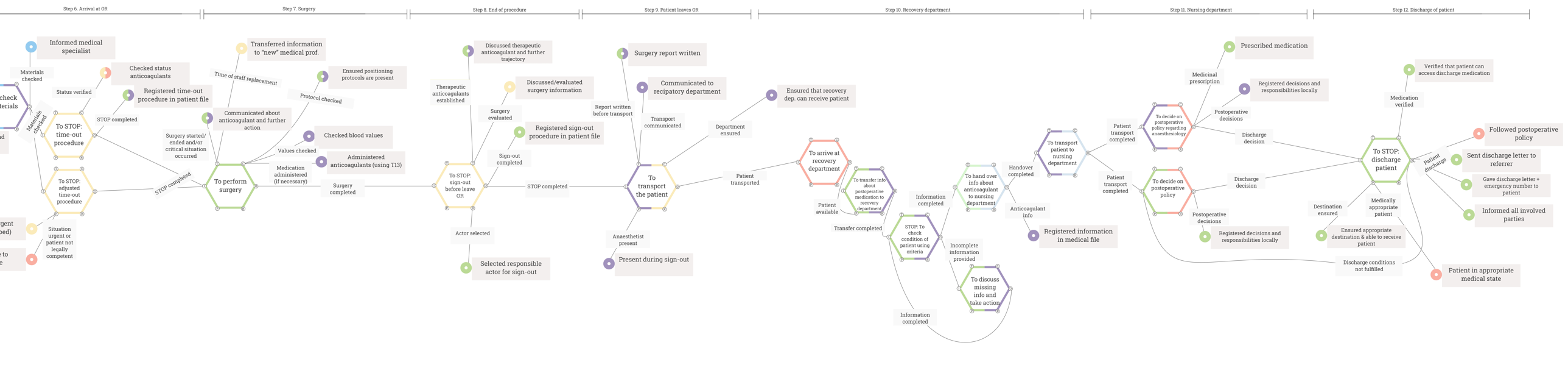

Supplement: mzaf074_Supplementary_Data [file mzaf074_supplementary_data.zip › Appendix E. Hospital 1 WAI_2_page-0001.jpg]

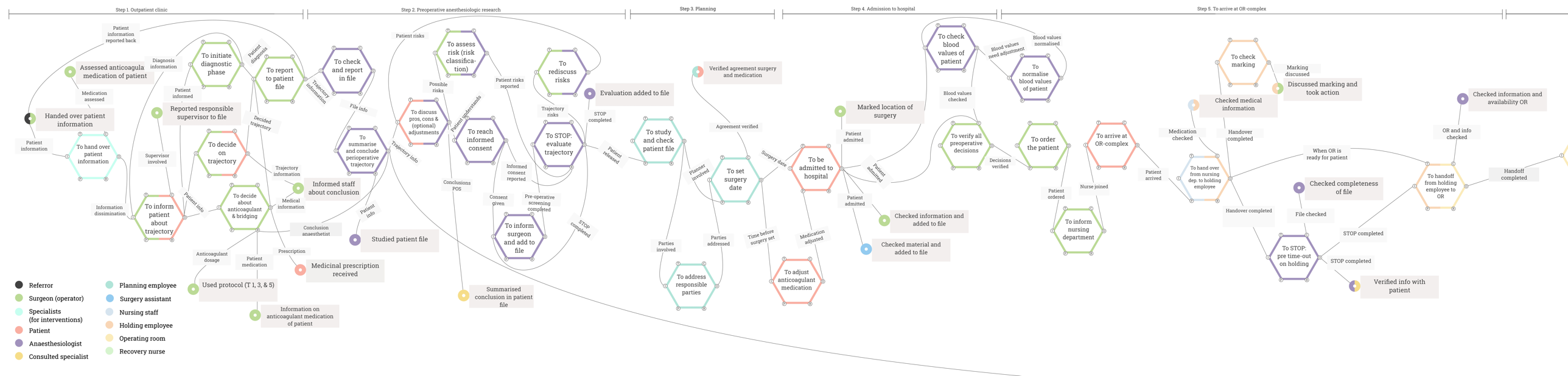

Supplement: mzaf074_Supplementary_Data [file mzaf074_supplementary_data.zip › Appendix E. Hospital 2 WAI_1_page-0001.jpg]

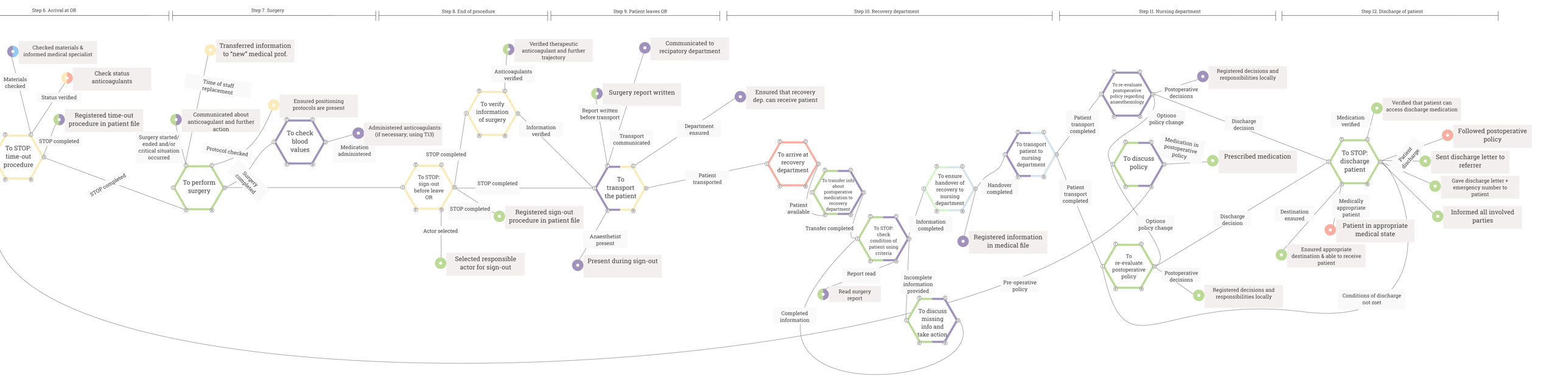

Supplement: mzaf074_Supplementary_Data [file mzaf074_supplementary_data.zip › Appendix E. Hospital 2 WAI_2_page-0001.jpg]

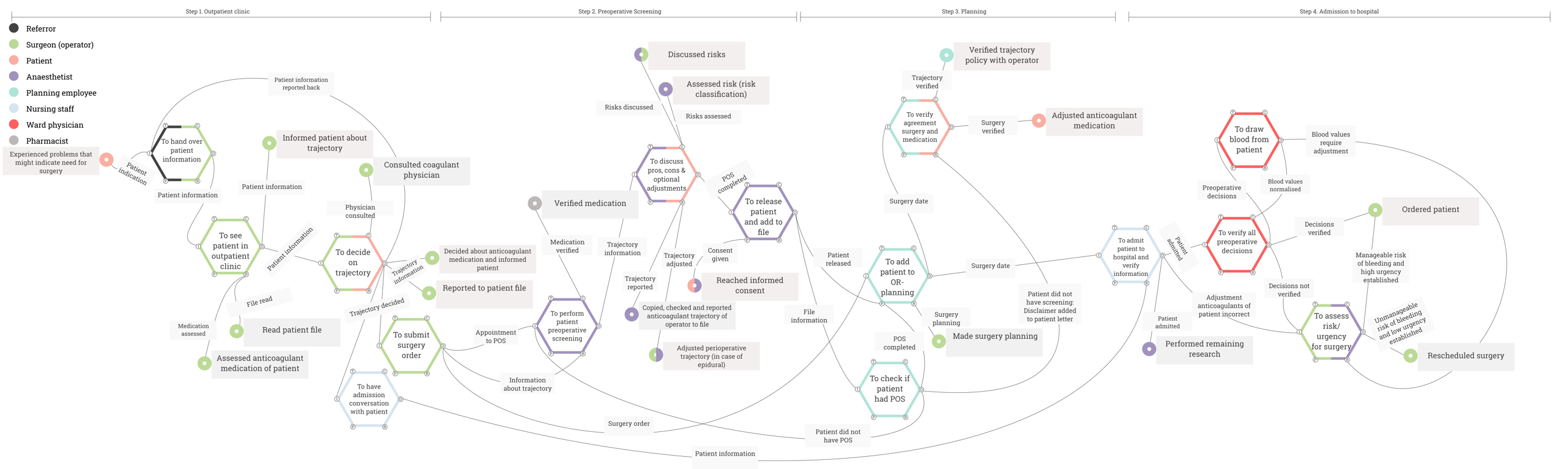

Supplement: mzaf074_Supplementary_Data [file mzaf074_supplementary_data.zip › Appendix F. Work-as-Done Hospital 1_page-0001.jpg]

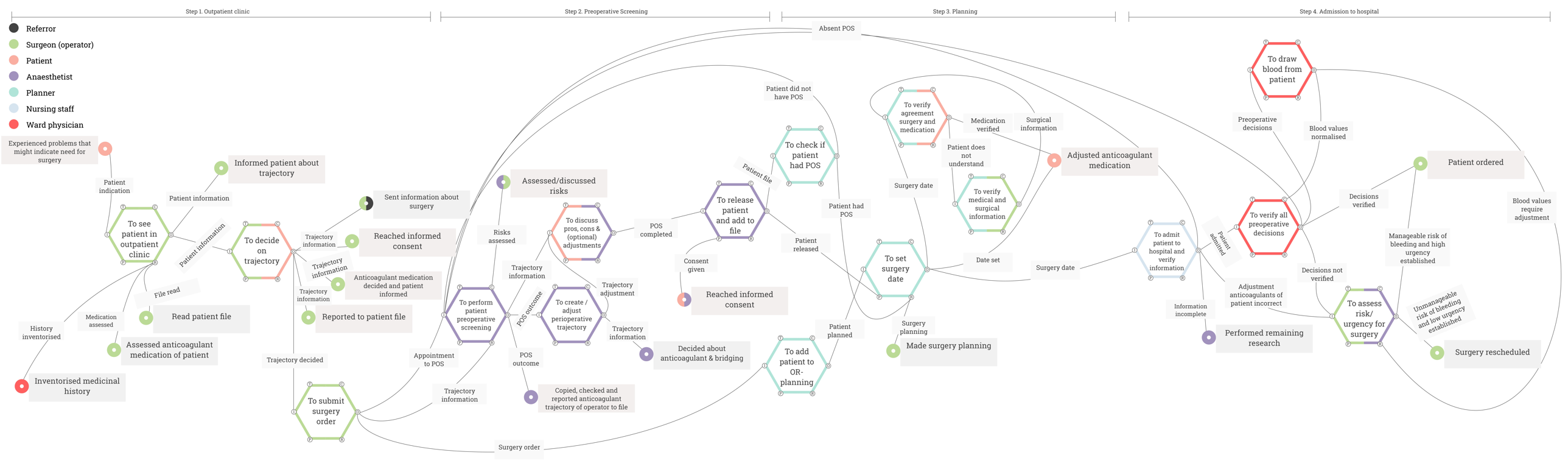

Supplement: mzaf074_Supplementary_Data [file mzaf074_supplementary_data.zip › Appendix F. Work-as-Done Hospital 2_page-0001.jpg]

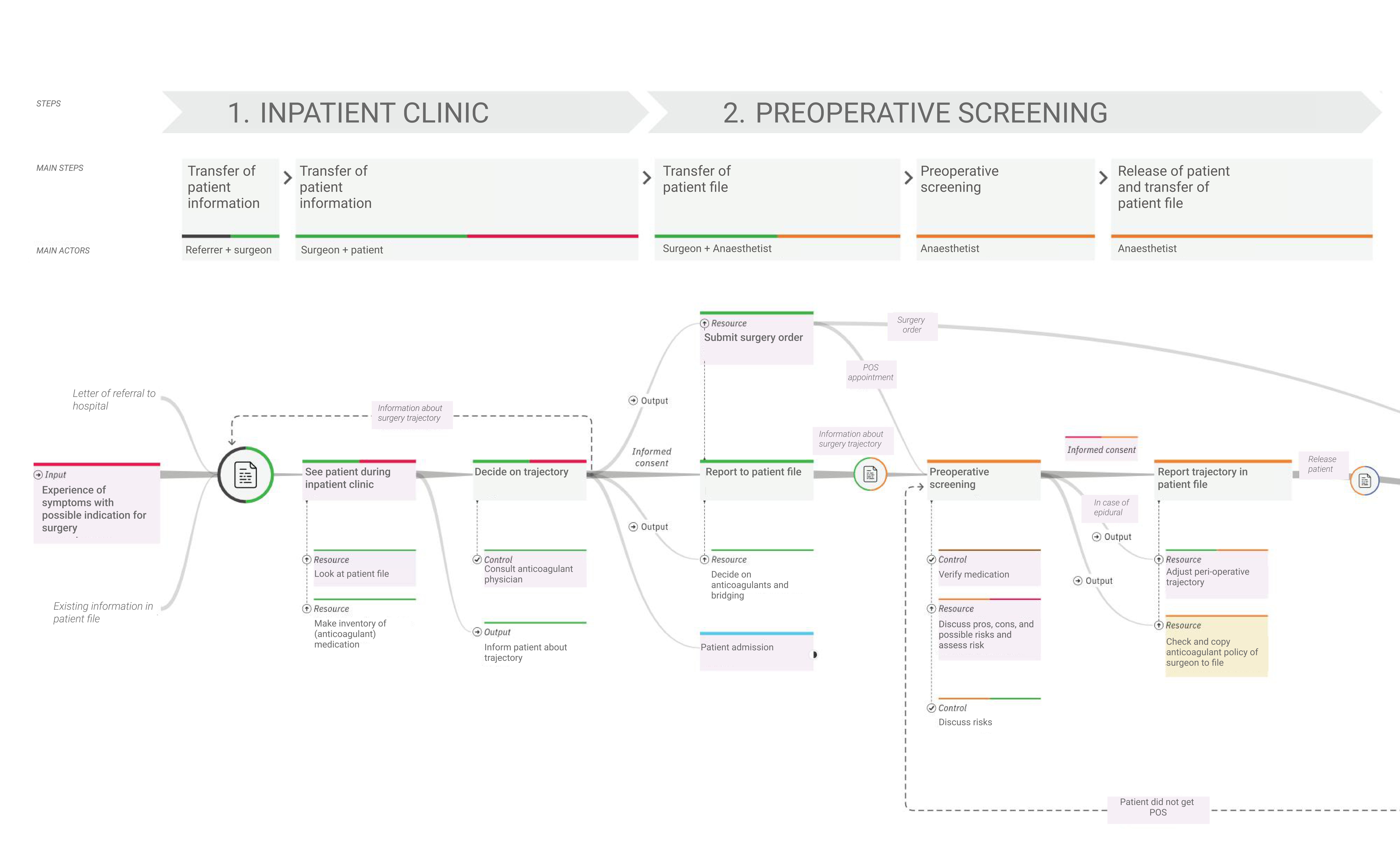

Supplement: mzaf074_Supplementary_Data [file mzaf074_supplementary_data.zip › Appendix G_Alternative visualisation_Work-as-Done_Hospital 1_1_page-0001.jpg]

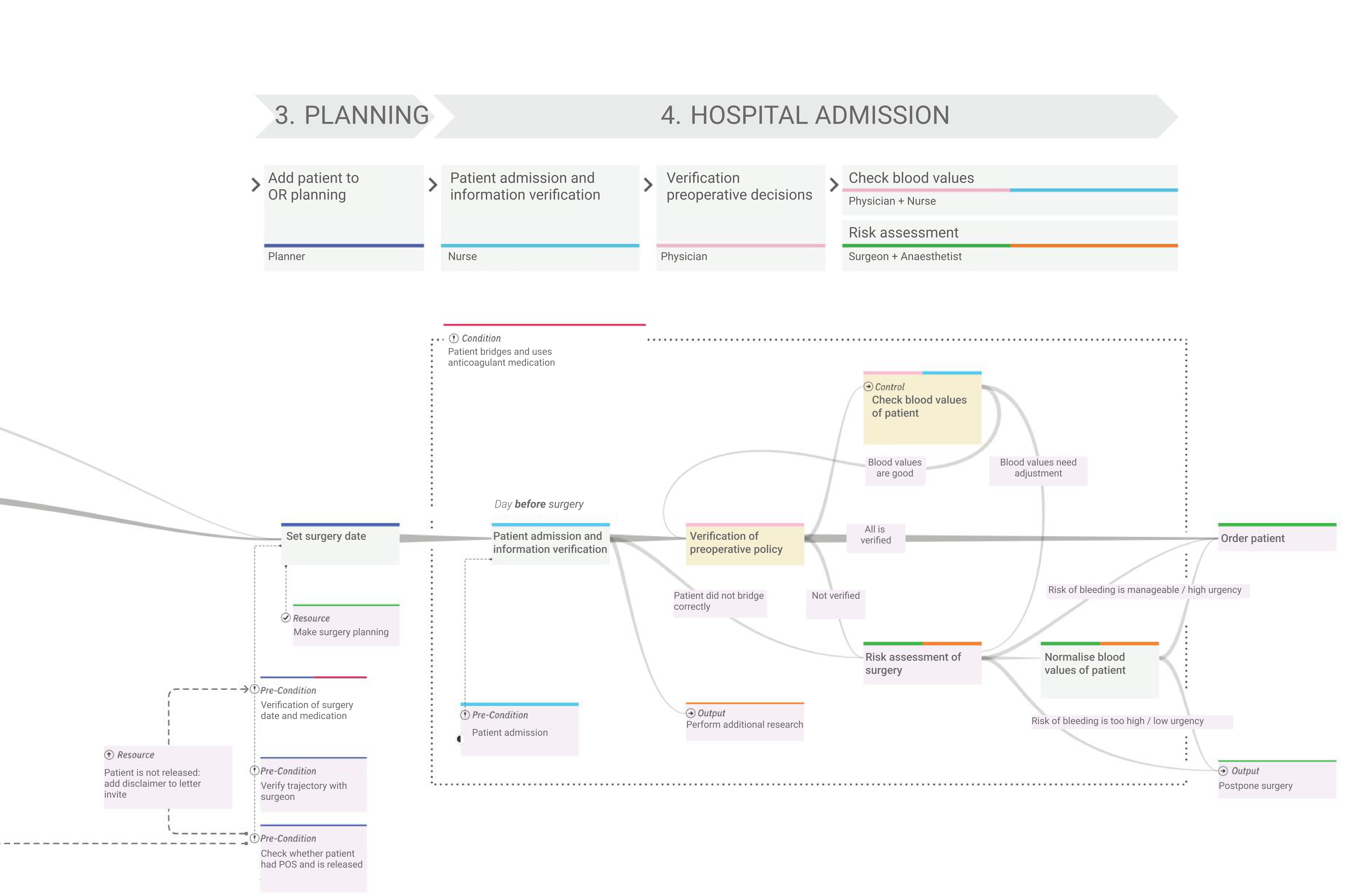

Supplement: mzaf074_Supplementary_Data [file mzaf074_supplementary_data.zip › Appendix G_Alternative visualisation_Work-as-Done_Hospital 1_2_page-0001.jpg]

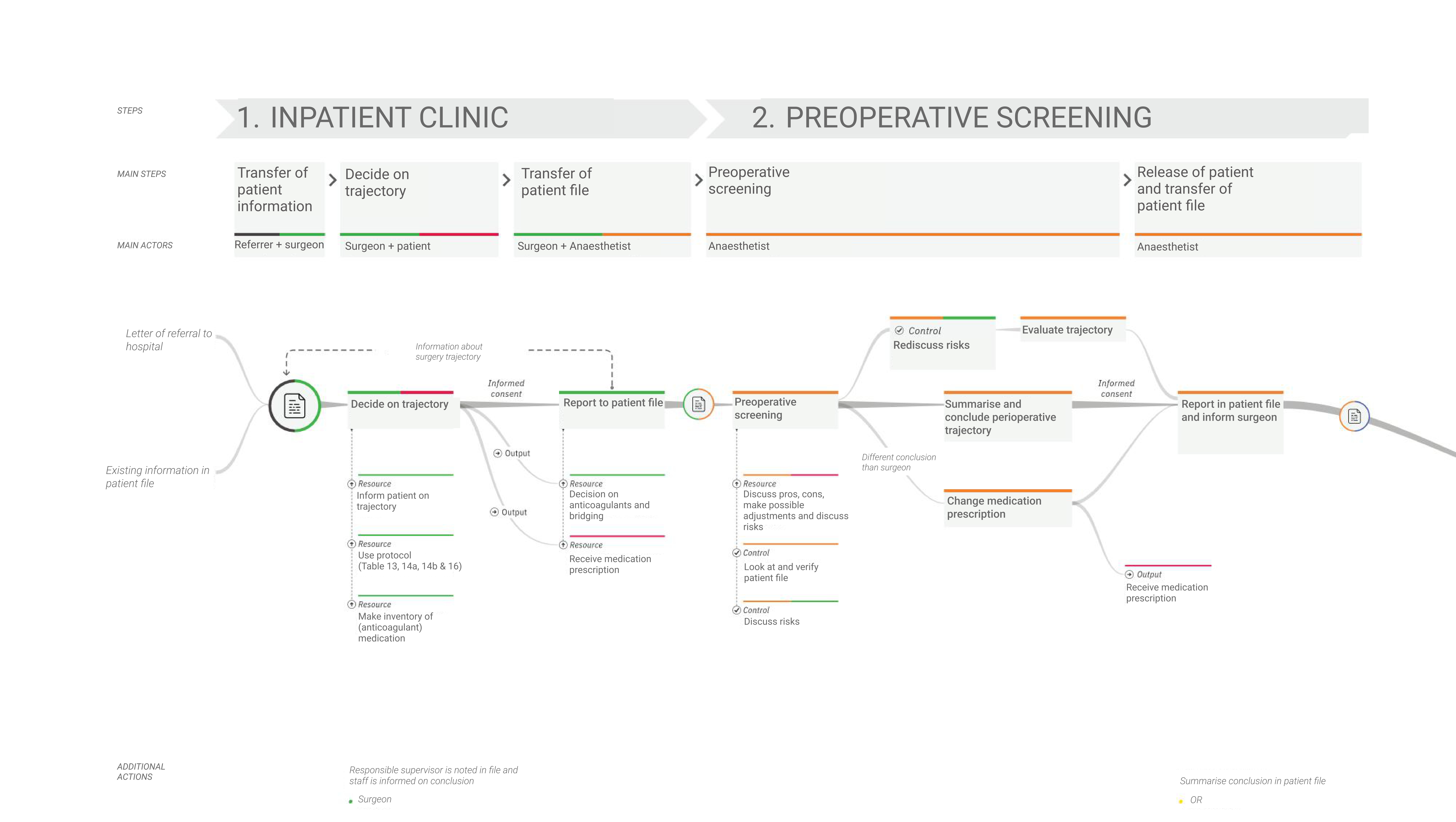

Supplement: mzaf074_Supplementary_Data [file mzaf074_supplementary_data.zip › Appendix G_Alternative visualisation_Work-as-Imagined_Hospital 1_1_page-0001.jpg]

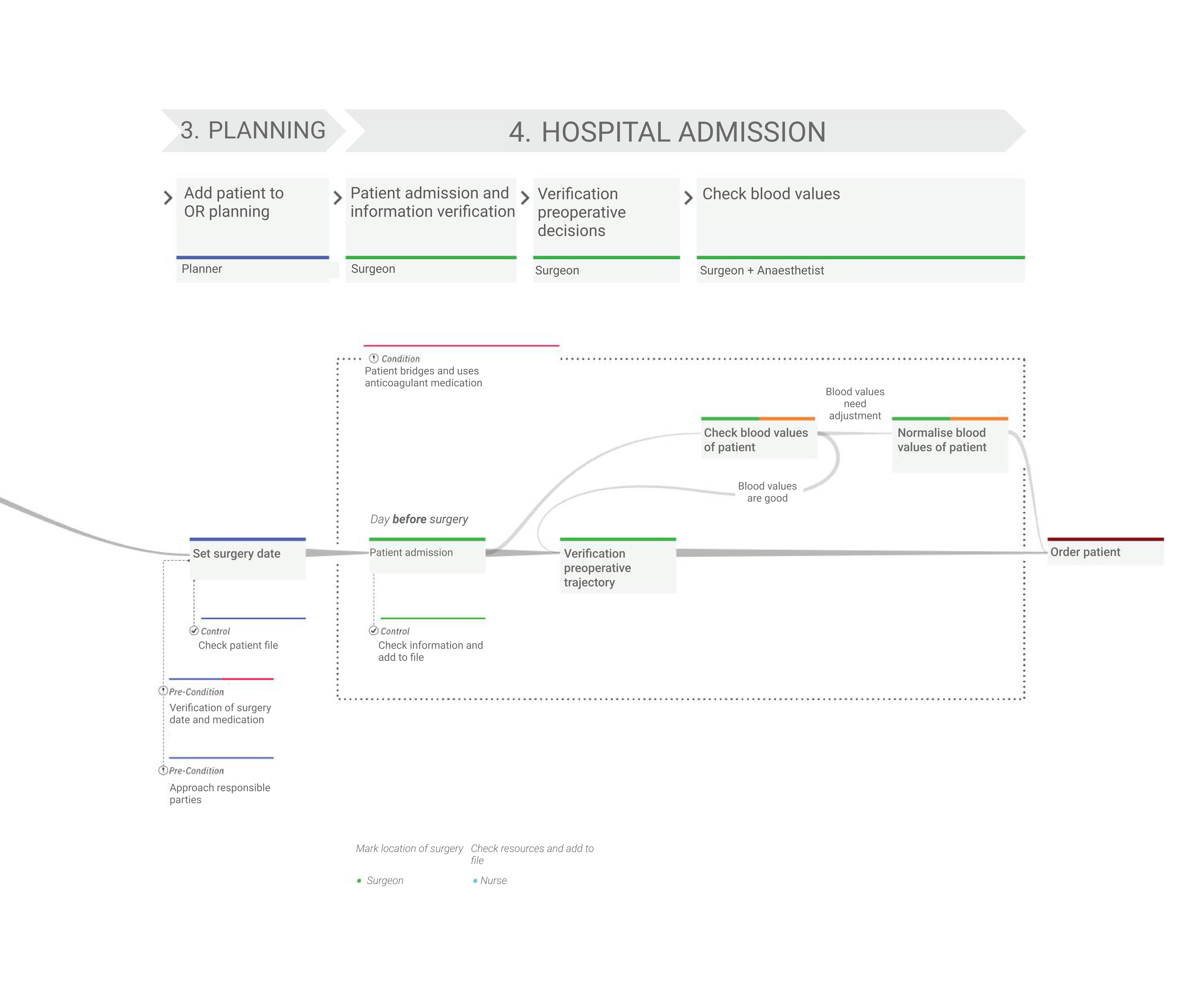

Supplement: mzaf074_Supplementary_Data [file mzaf074_supplementary_data.zip › Appendix G_Alternative visualisation_Work-as-Imagined_Hospital 1_2_page-0001.jpg]
